# Supplementary figures and images for: Quantitative research on the efficiency of ancient information transmission system: A case study of Wenzhou in the Ming Dynasty
Source: PLoS One. 2021 Apr 23;16(4):e0250622. doi: 10.1371/journal.pone.0250622 (PMC8064551; doi:10.1371/journal.pone.0250622)

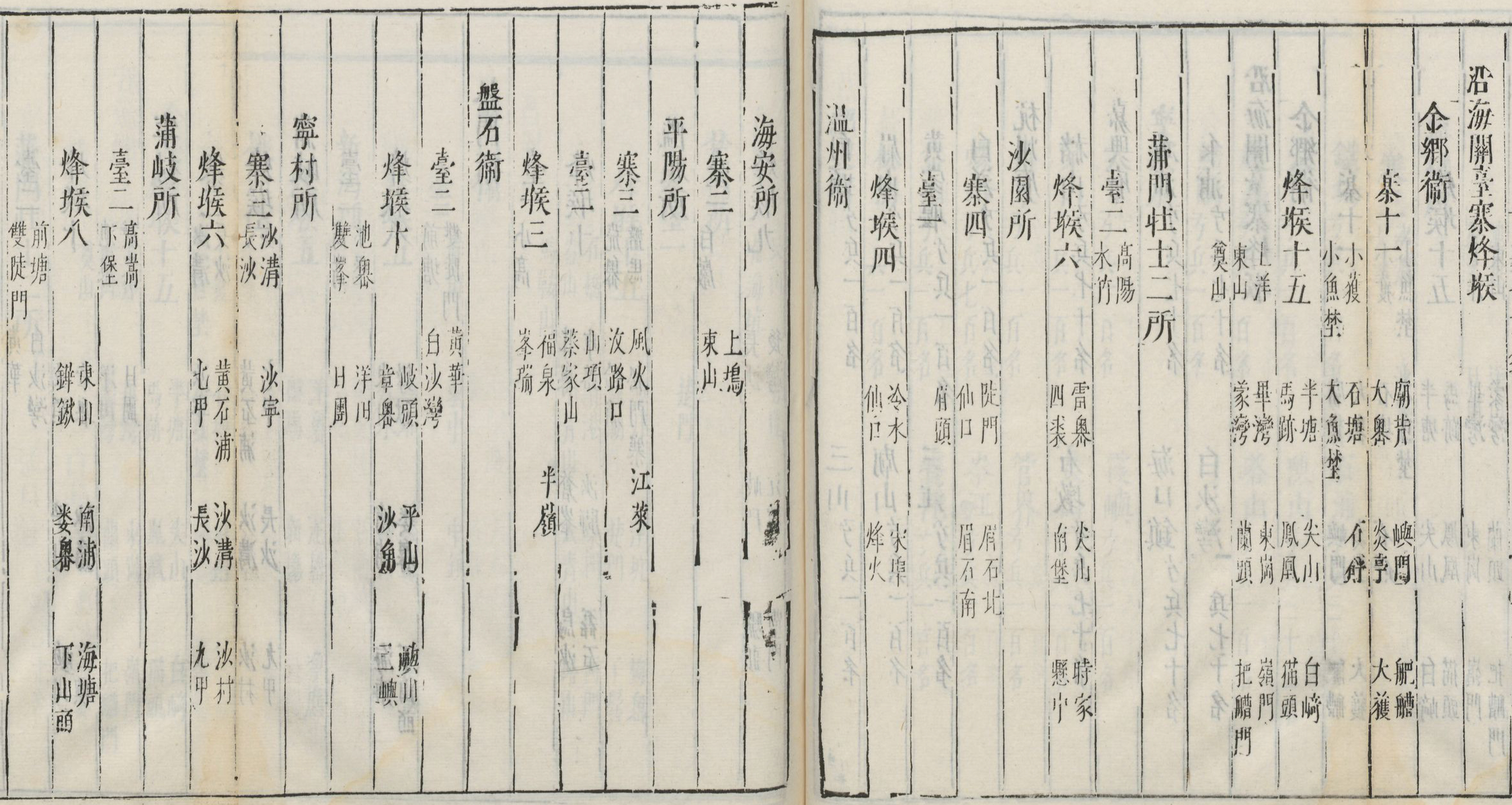

Supplement: S1 File — (TIF) [file pone.0250622.s001.tif]

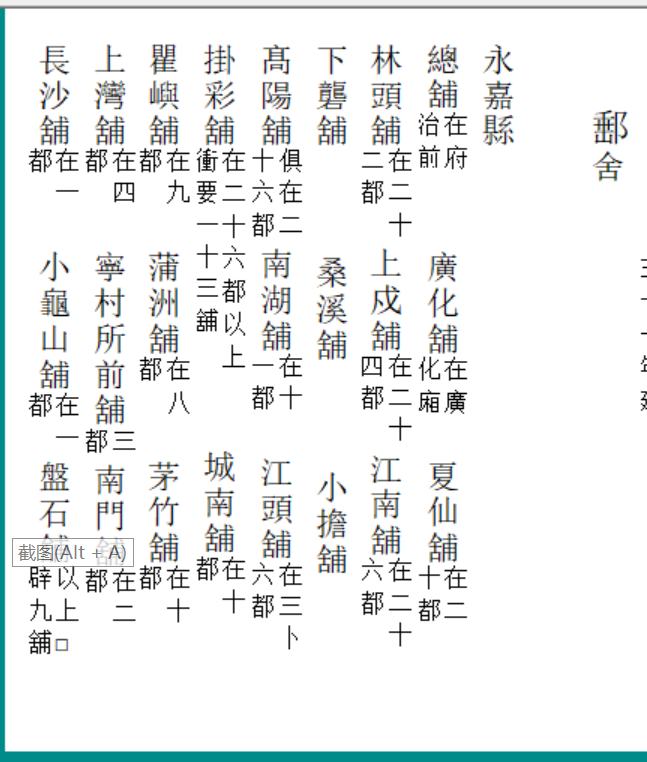

Supplement: S3 File — (ZIP) [file pone.0250622.s003.zip › S1 Scanned version of local chornicles/万历温州府志1.png]

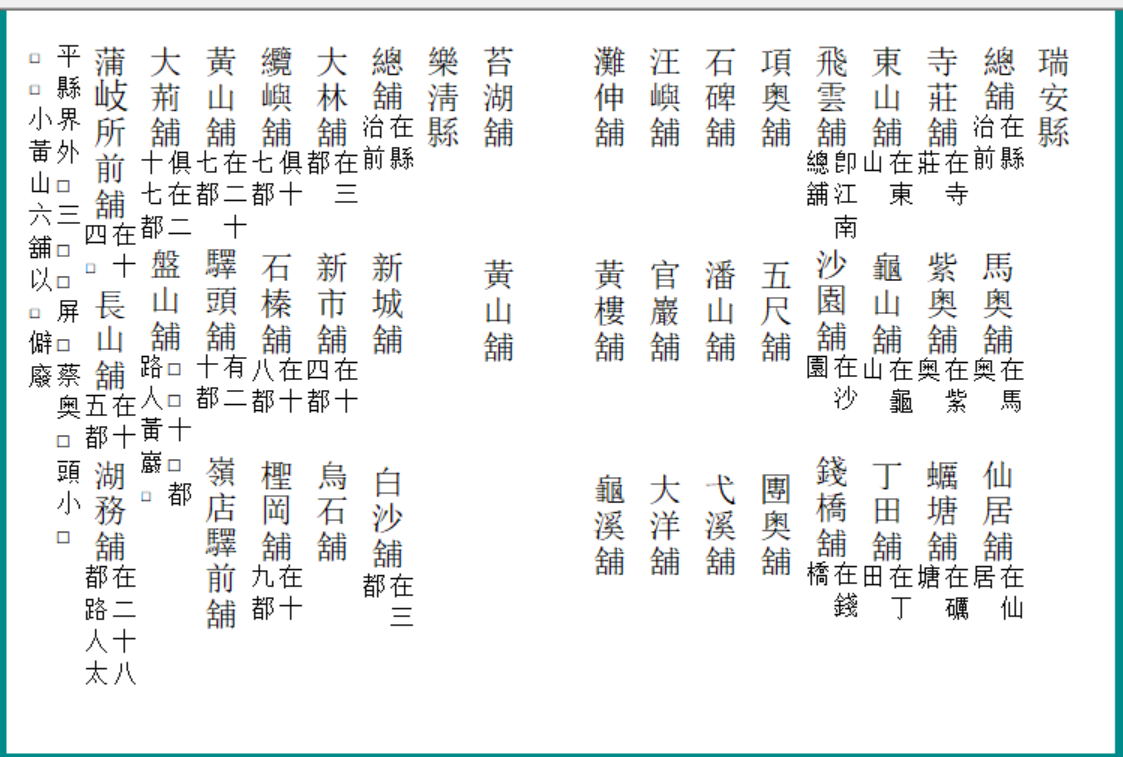

Supplement: S3 File — (ZIP) [file pone.0250622.s003.zip › S1 Scanned version of local chornicles/万历温州府志2.png]

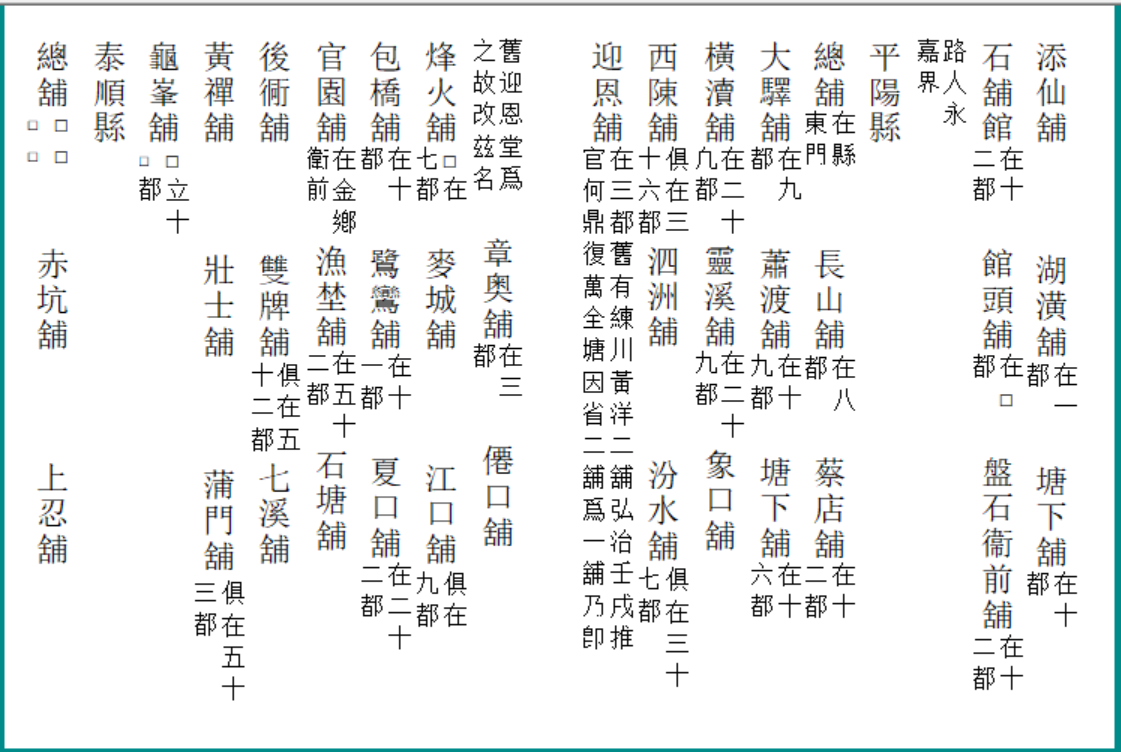

Supplement: S3 File — (ZIP) [file pone.0250622.s003.zip › S1 Scanned version of local chornicles/万历温州府志3.png]

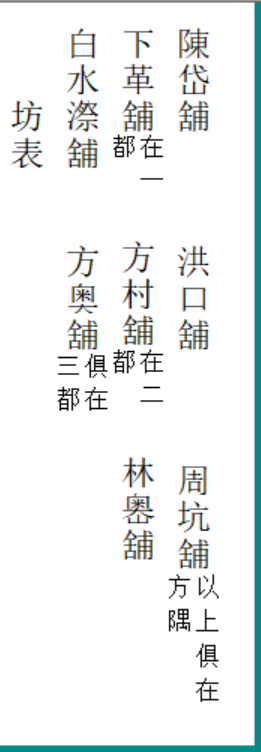

Supplement: S3 File — (ZIP) [file pone.0250622.s003.zip › S1 Scanned version of local chornicles/万历温州府志4.png]

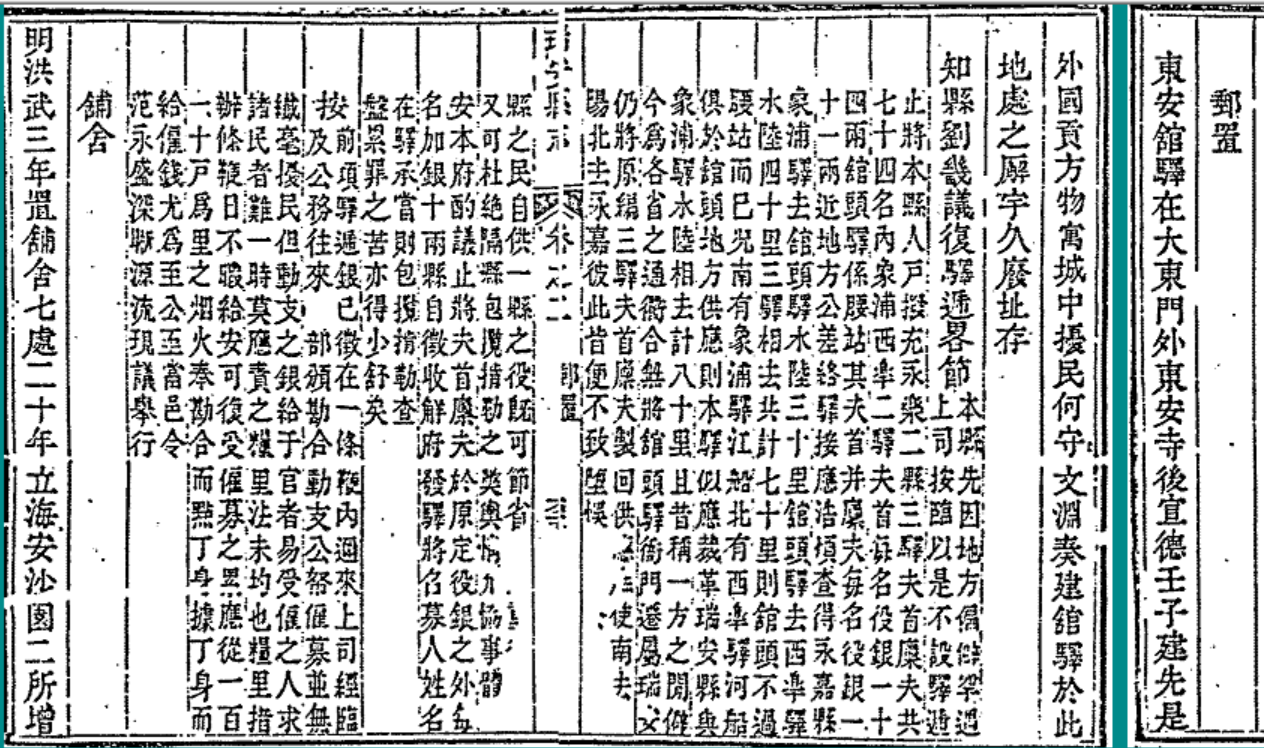

Supplement: S3 File — (ZIP) [file pone.0250622.s003.zip › S1 Scanned version of local chornicles/乾隆瑞安县志1.png]

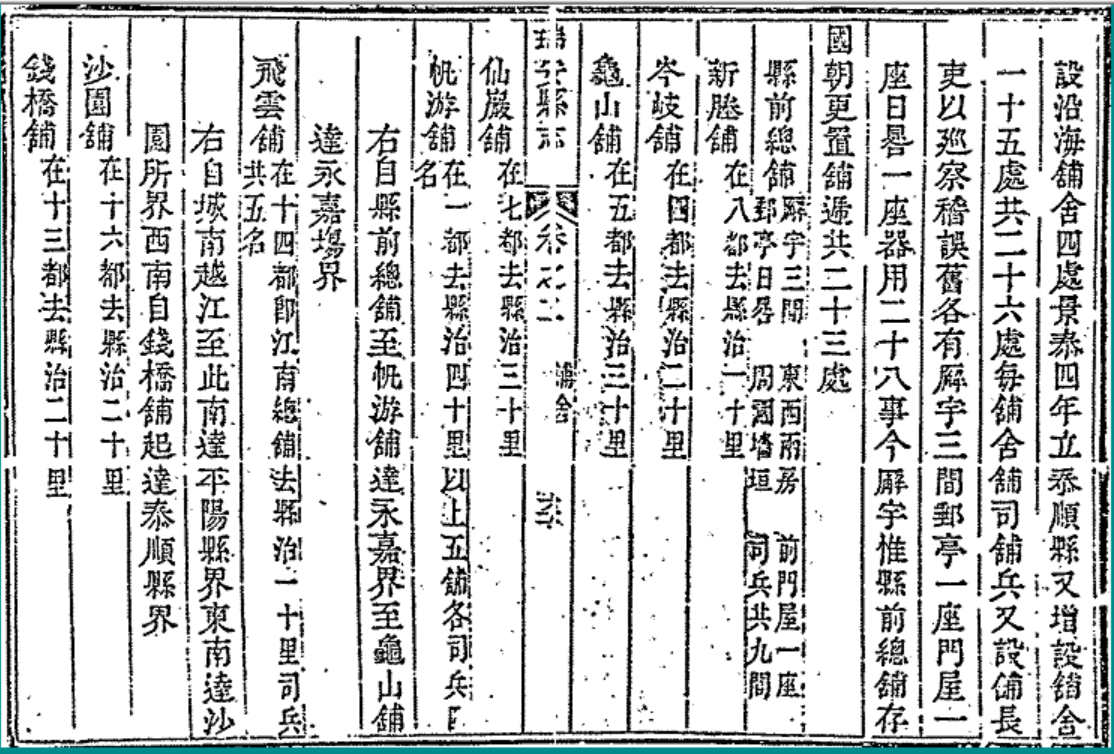

Supplement: S3 File — (ZIP) [file pone.0250622.s003.zip › S1 Scanned version of local chornicles/乾隆瑞安县志2.png]

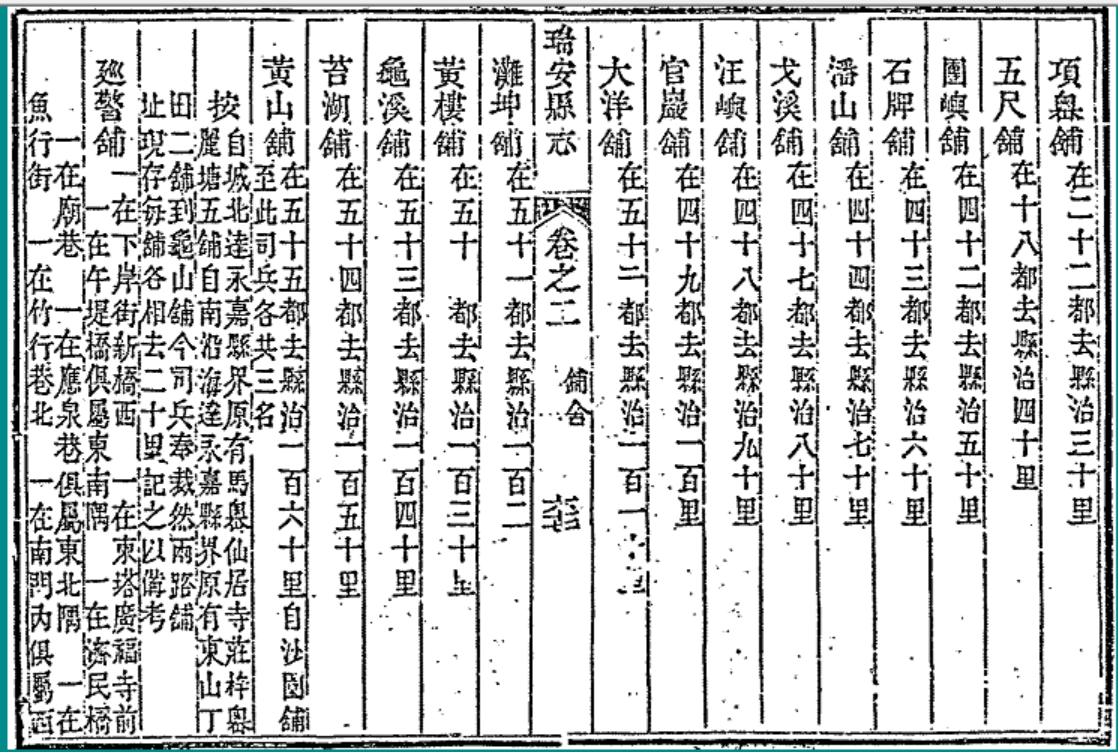

Supplement: S3 File — (ZIP) [file pone.0250622.s003.zip › S1 Scanned version of local chornicles/乾隆瑞安县志3.png]

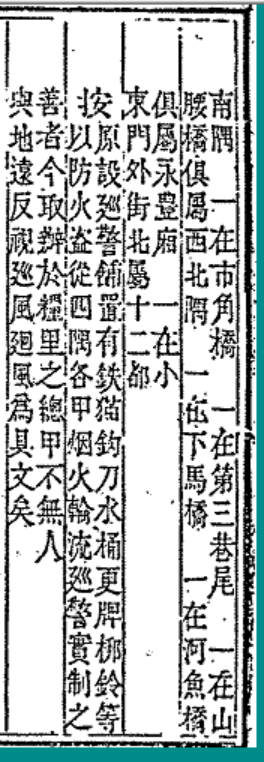

Supplement: S3 File — (ZIP) [file pone.0250622.s003.zip › S1 Scanned version of local chornicles/乾隆瑞安县志4.png]

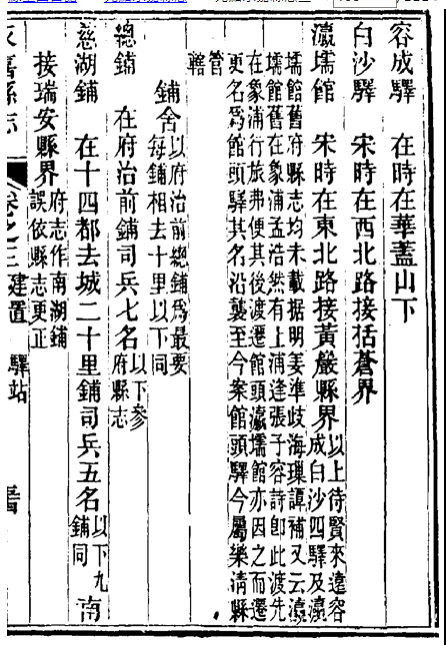

Supplement: S3 File — (ZIP) [file pone.0250622.s003.zip › S1 Scanned version of local chornicles/光绪永嘉县志1.png]

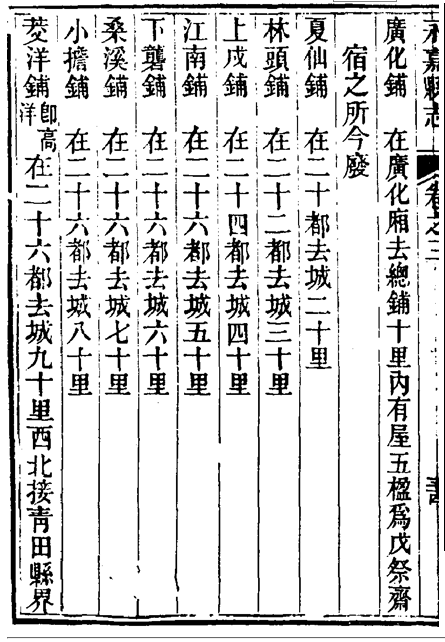

Supplement: S3 File — (ZIP) [file pone.0250622.s003.zip › S1 Scanned version of local chornicles/光绪永嘉县志2.png]

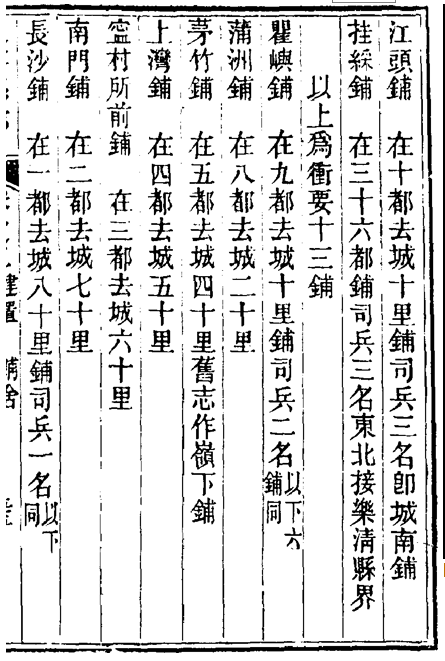

Supplement: S3 File — (ZIP) [file pone.0250622.s003.zip › S1 Scanned version of local chornicles/光绪永嘉县志3.png]

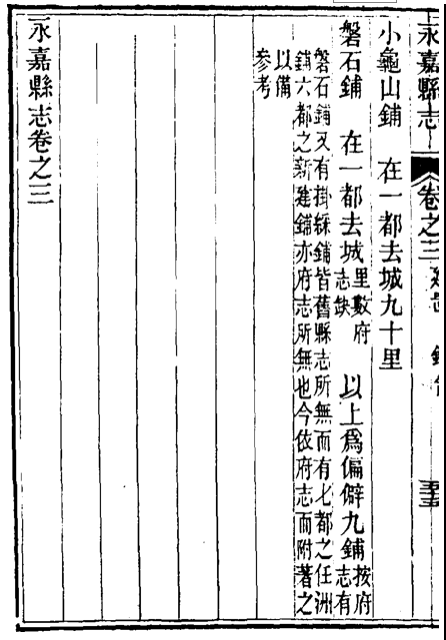

Supplement: S3 File — (ZIP) [file pone.0250622.s003.zip › S1 Scanned version of local chornicles/光绪永嘉县志4.png]

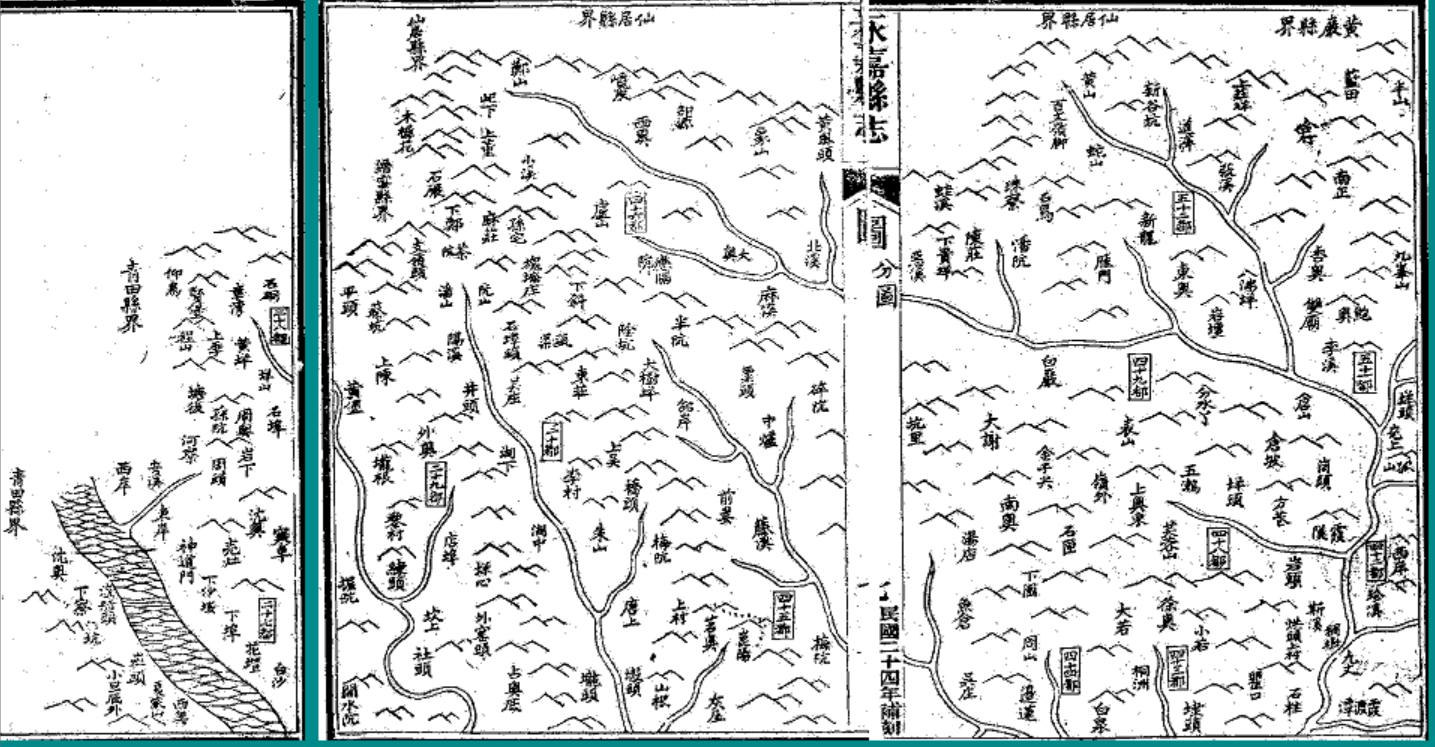

Supplement: S3 File — (ZIP) [file pone.0250622.s003.zip › S1 Scanned version of local chornicles/光绪永嘉县志5.png]

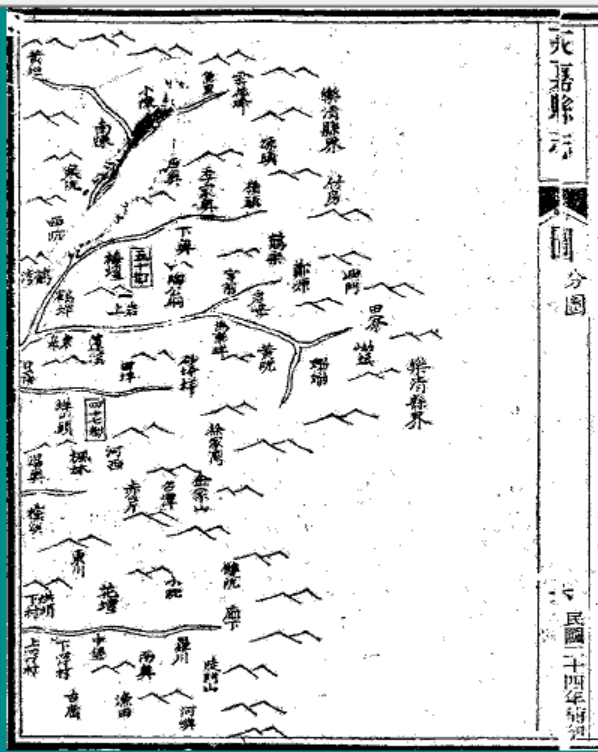

Supplement: S3 File — (ZIP) [file pone.0250622.s003.zip › S1 Scanned version of local chornicles/光绪永嘉县志6.png]

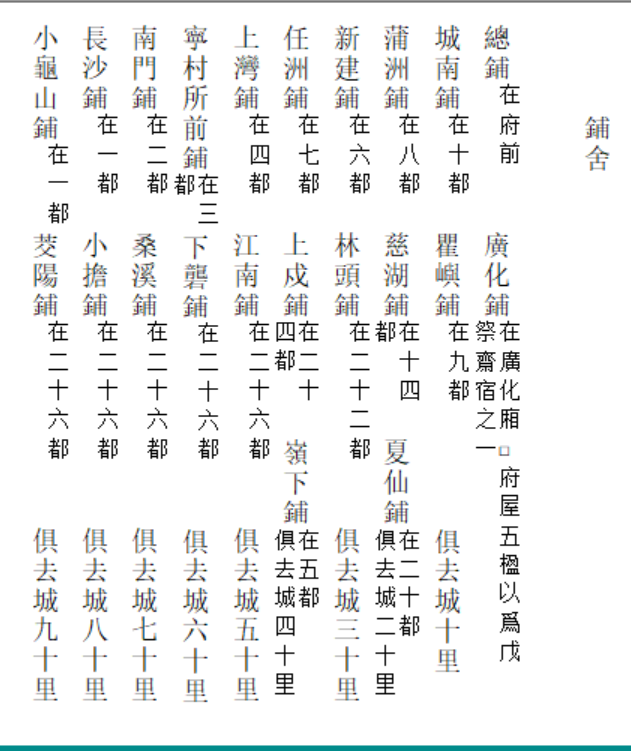

Supplement: S3 File — (ZIP) [file pone.0250622.s003.zip › S1 Scanned version of local chornicles/嘉靖永嘉县志1.png]

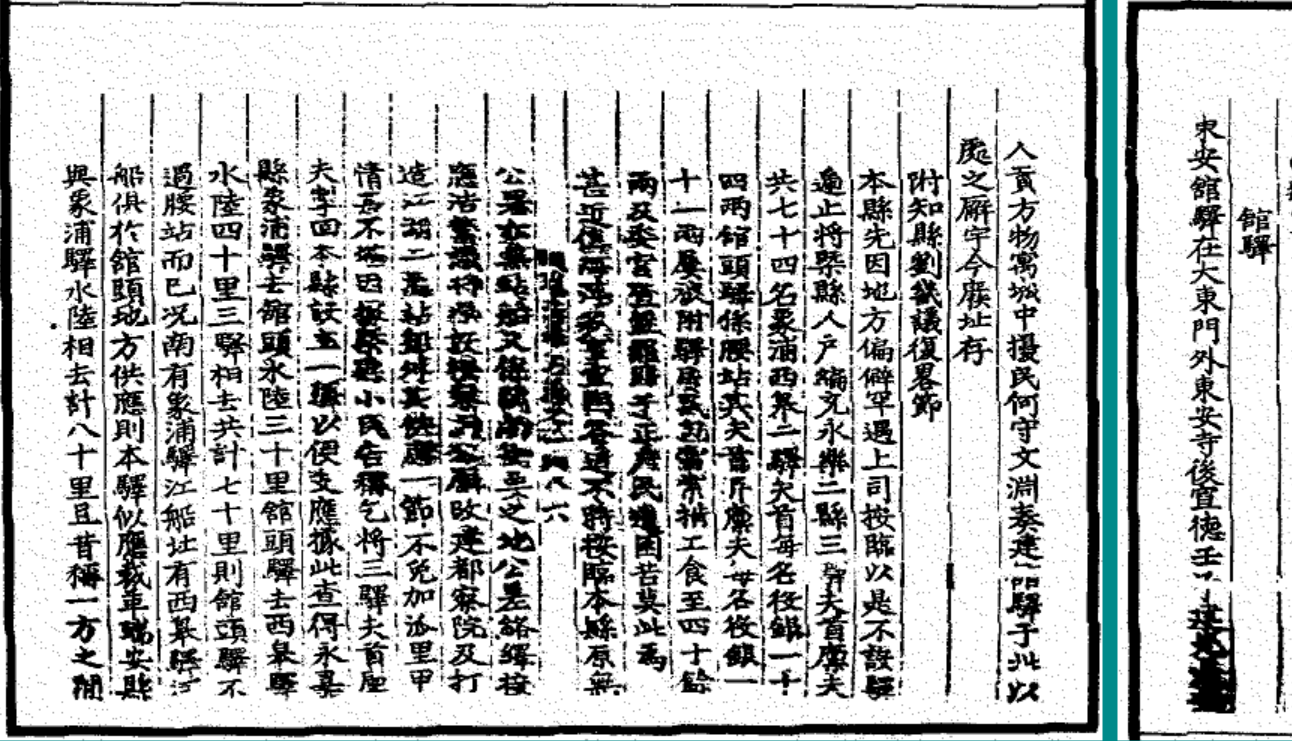

Supplement: S3 File — (ZIP) [file pone.0250622.s003.zip › S1 Scanned version of local chornicles/嘉靖瑞安县志1.png]

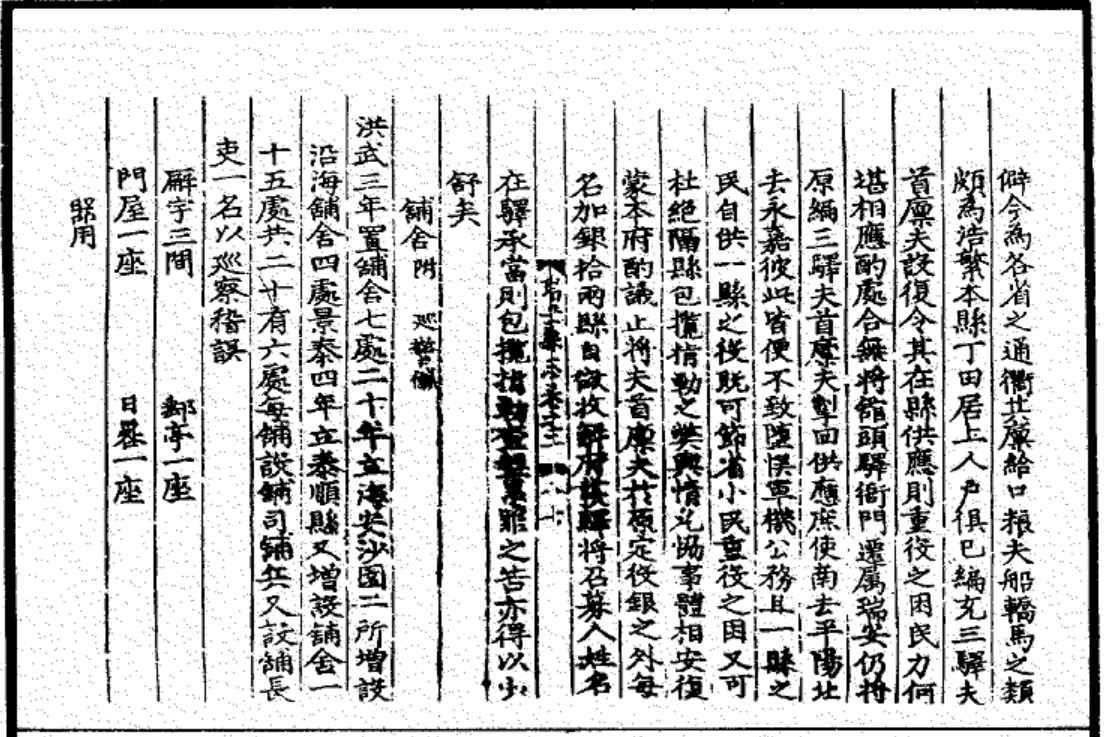

Supplement: S3 File — (ZIP) [file pone.0250622.s003.zip › S1 Scanned version of local chornicles/嘉靖瑞安县志2.png]

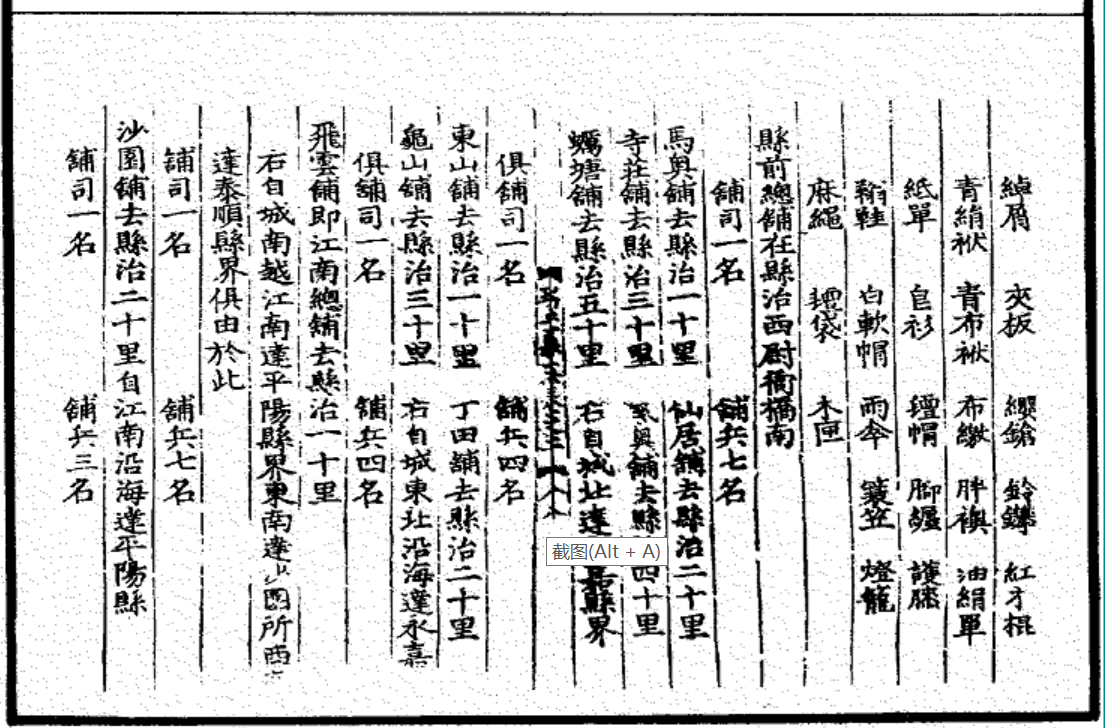

Supplement: S3 File — (ZIP) [file pone.0250622.s003.zip › S1 Scanned version of local chornicles/嘉靖瑞安县志3.png]

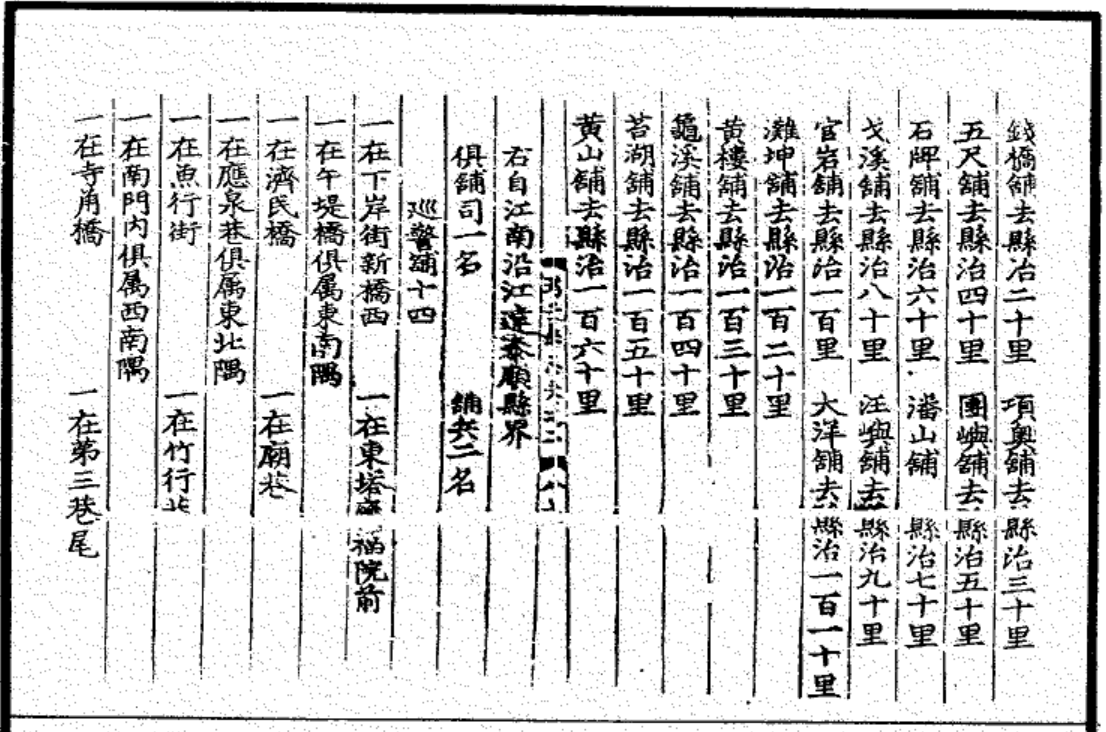

Supplement: S3 File — (ZIP) [file pone.0250622.s003.zip › S1 Scanned version of local chornicles/嘉靖瑞安县志4.png]

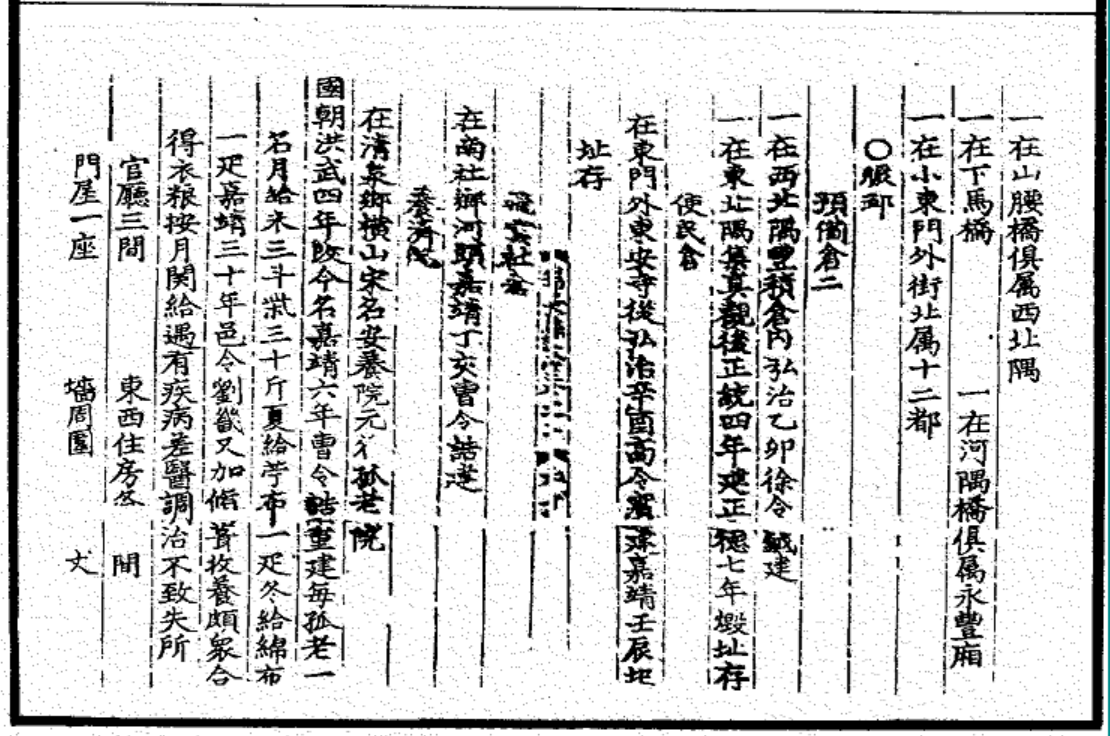

Supplement: S3 File — (ZIP) [file pone.0250622.s003.zip › S1 Scanned version of local chornicles/嘉靖瑞安县志5.png]

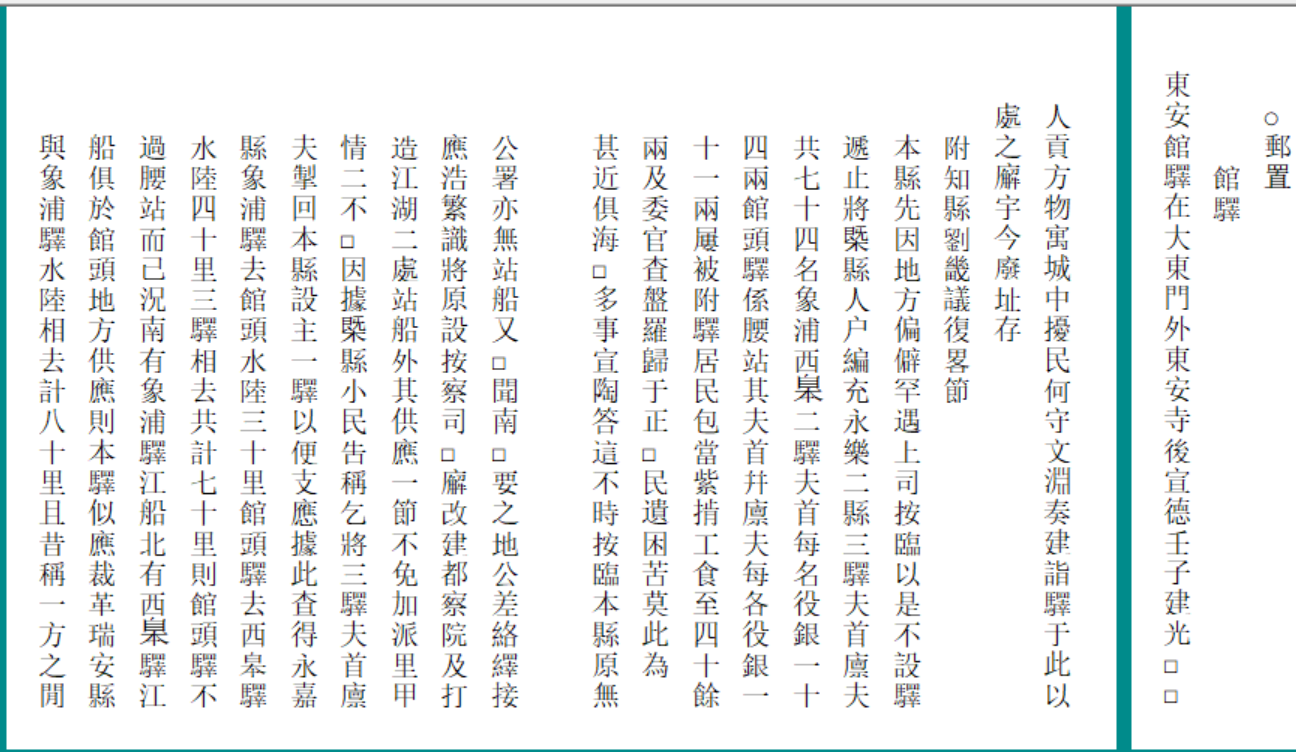

Supplement: S3 File — (ZIP) [file pone.0250622.s003.zip › S1 Scanned version of local chornicles/嘉靖瑞安县志6.png]

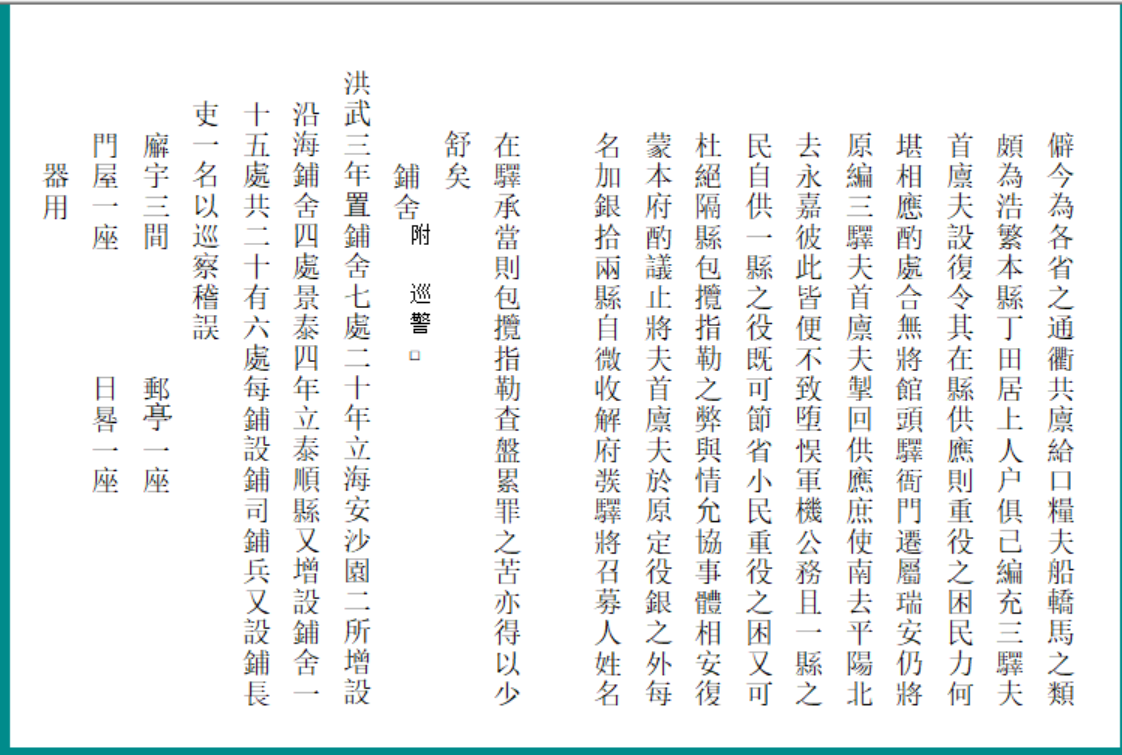

Supplement: S3 File — (ZIP) [file pone.0250622.s003.zip › S1 Scanned version of local chornicles/嘉靖瑞安县志7.png]

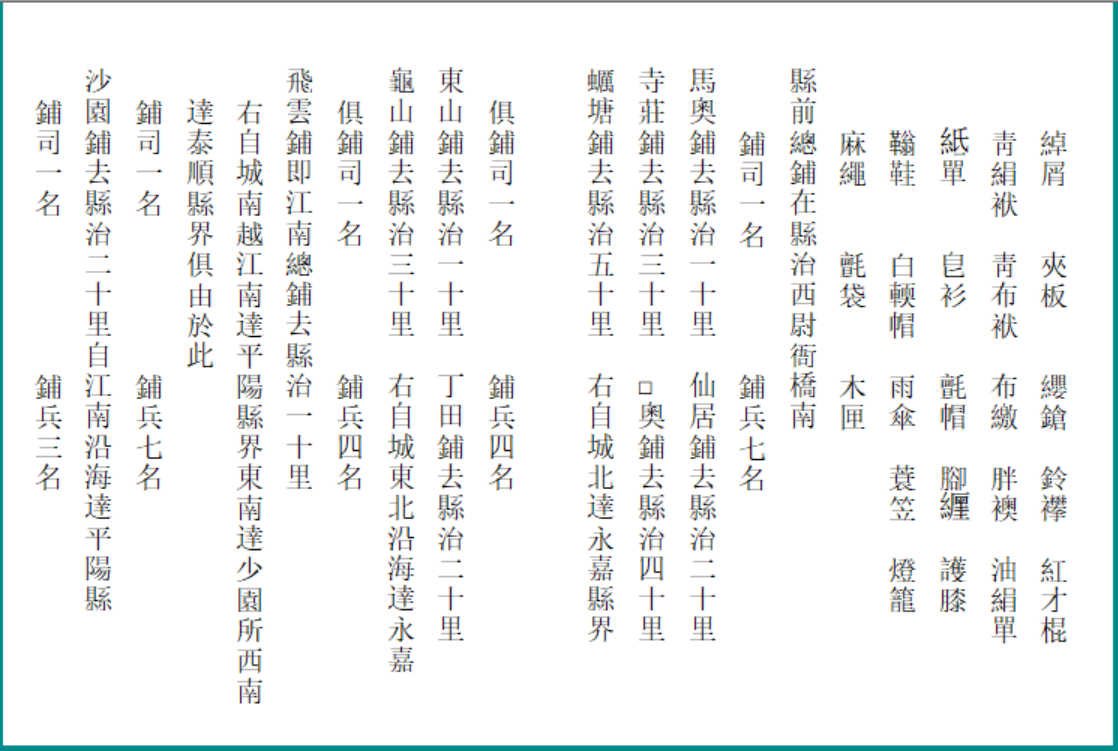

Supplement: S3 File — (ZIP) [file pone.0250622.s003.zip › S1 Scanned version of local chornicles/嘉靖瑞安县志8.png]

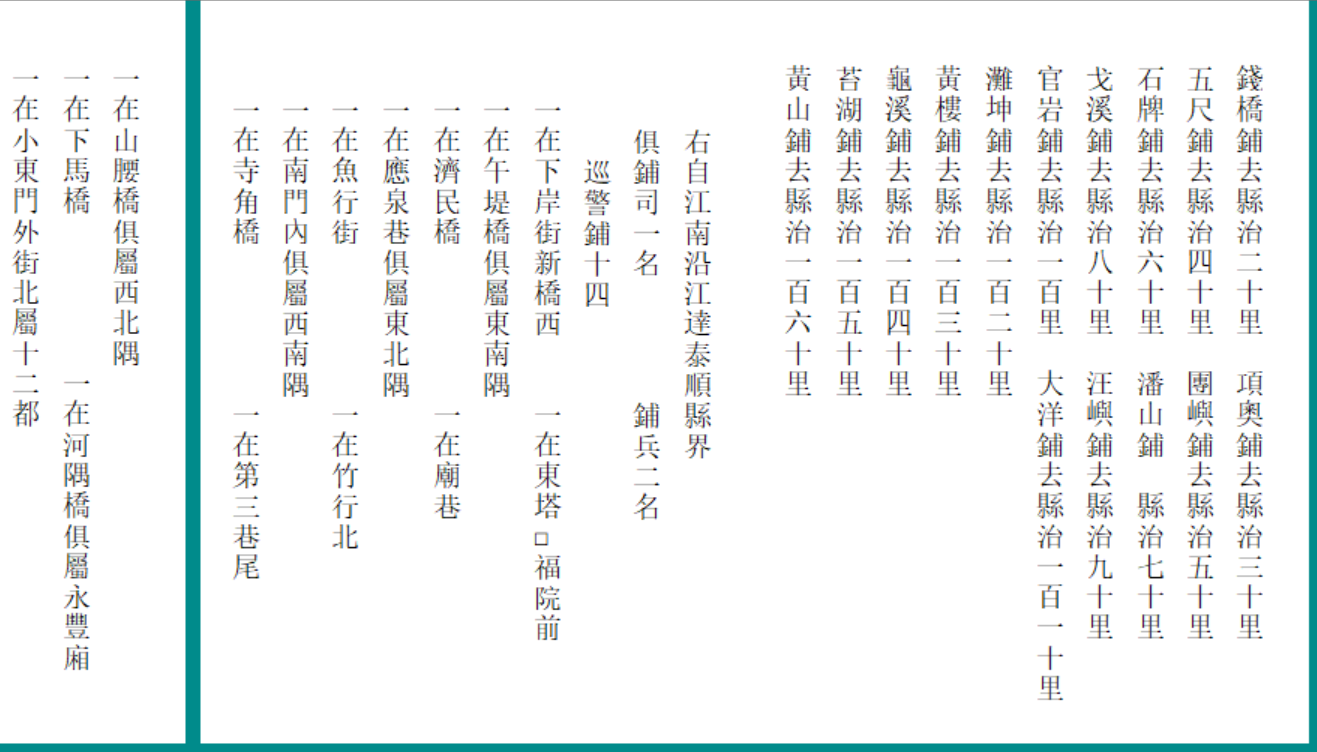

Supplement: S3 File — (ZIP) [file pone.0250622.s003.zip › S1 Scanned version of local chornicles/嘉靖瑞安县志9.png]

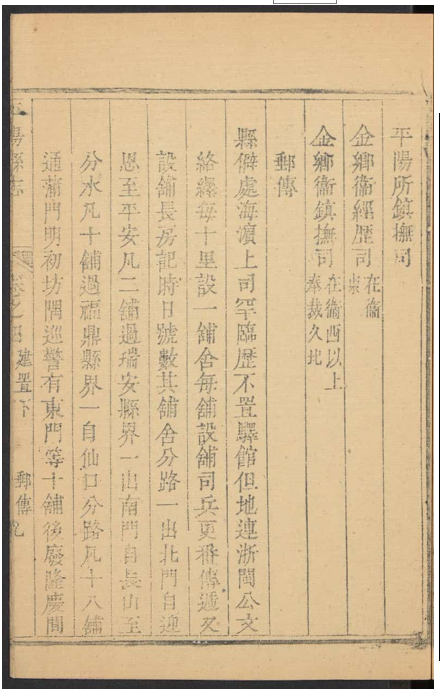

Supplement: S3 File — (ZIP) [file pone.0250622.s003.zip › S1 Scanned version of local chornicles/平阳县志1.png]

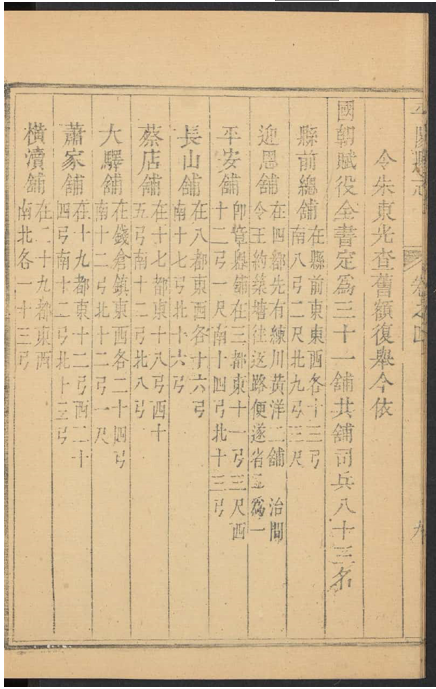

Supplement: S3 File — (ZIP) [file pone.0250622.s003.zip › S1 Scanned version of local chornicles/平阳县志2.png]

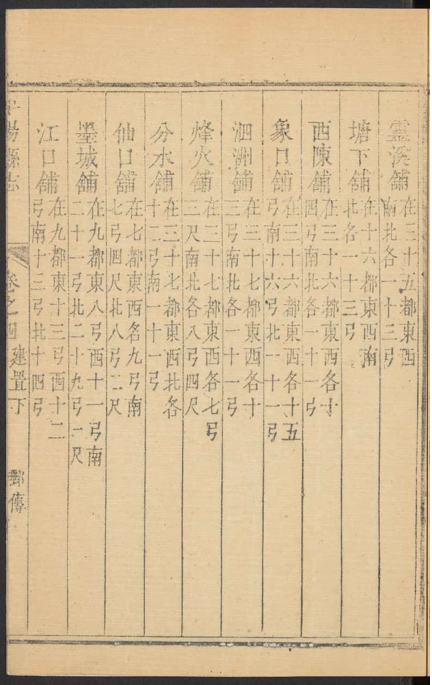

Supplement: S3 File — (ZIP) [file pone.0250622.s003.zip › S1 Scanned version of local chornicles/平阳县志3.png]

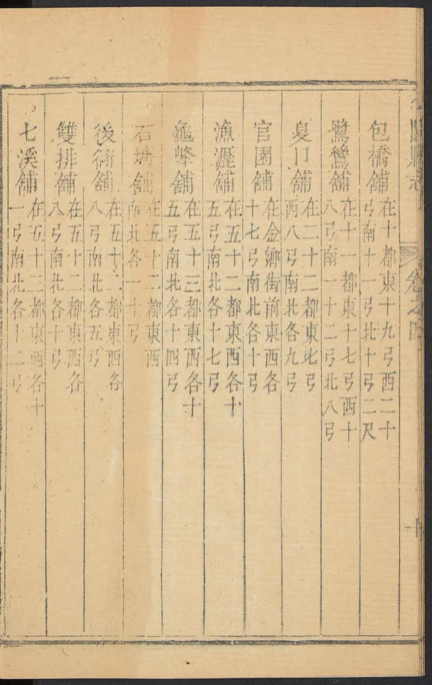

Supplement: S3 File — (ZIP) [file pone.0250622.s003.zip › S1 Scanned version of local chornicles/平阳县志4.png]

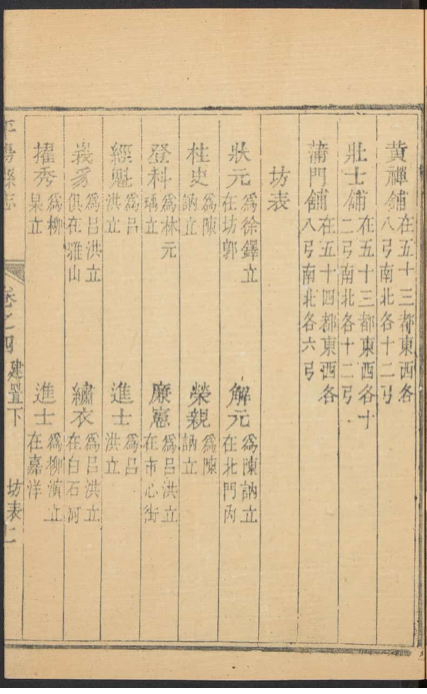

Supplement: S3 File — (ZIP) [file pone.0250622.s003.zip › S1 Scanned version of local chornicles/平阳县志5.png]

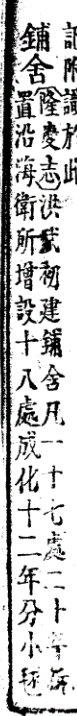

Supplement: S3 File — (ZIP) [file pone.0250622.s003.zip › S1 Scanned version of local chornicles/微信图片_20200324153455.png]

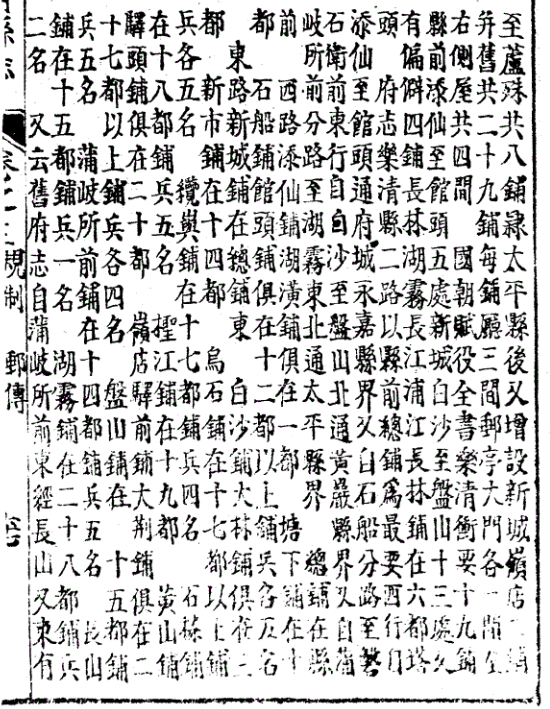

Supplement: S3 File — (ZIP) [file pone.0250622.s003.zip › S1 Scanned version of local chornicles/微信图片_20200324153547.png]

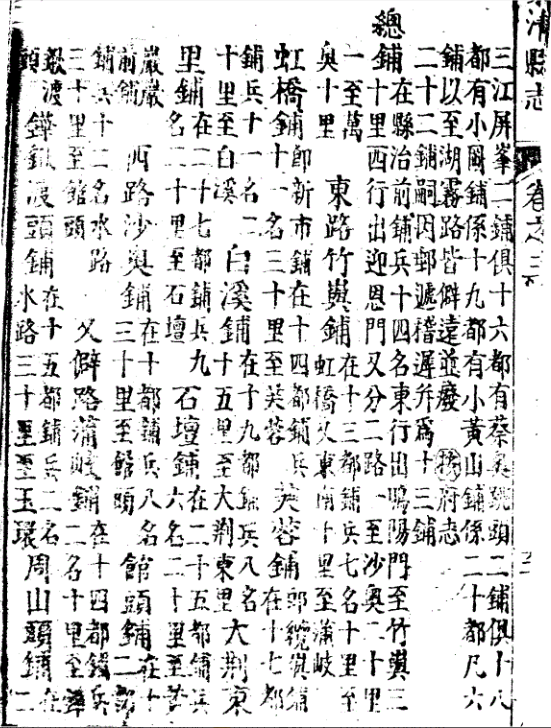

Supplement: S3 File — (ZIP) [file pone.0250622.s003.zip › S1 Scanned version of local chornicles/微信图片_20200324153551.png]

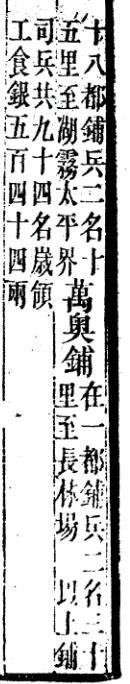

Supplement: S3 File — (ZIP) [file pone.0250622.s003.zip › S1 Scanned version of local chornicles/微信图片_20200324153555.png]

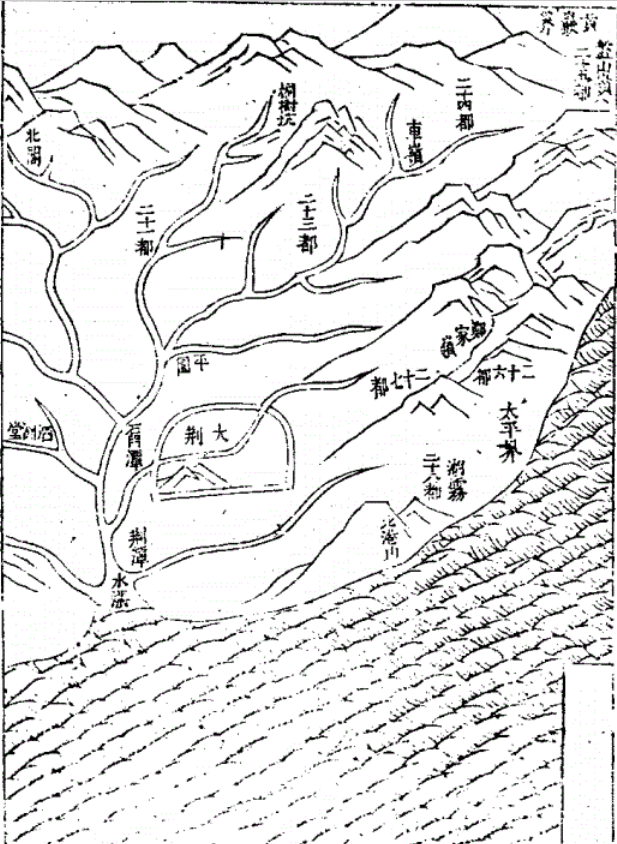

Supplement: S3 File — (ZIP) [file pone.0250622.s003.zip › S1 Scanned version of local chornicles/微信图片_20200324153558.png]

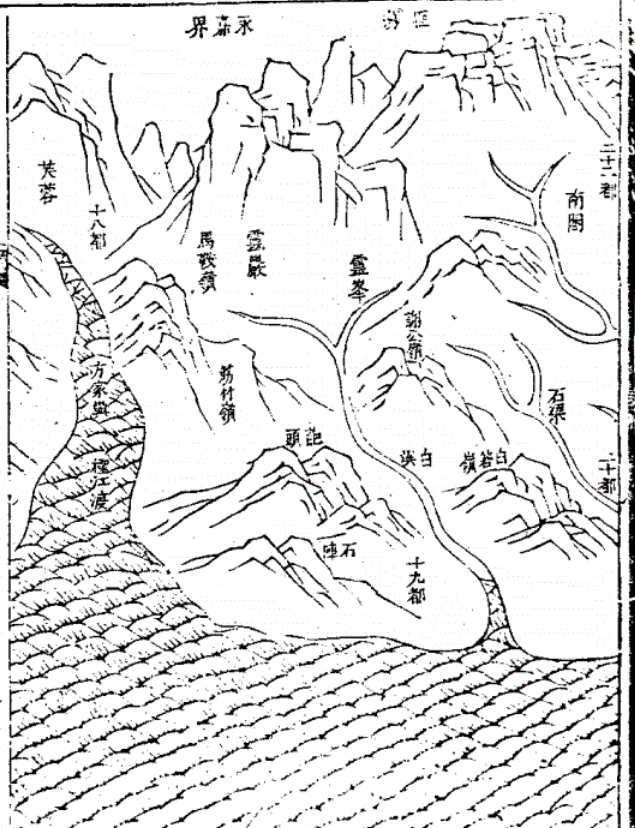

Supplement: S3 File — (ZIP) [file pone.0250622.s003.zip › S1 Scanned version of local chornicles/微信图片_20200324153602.png]

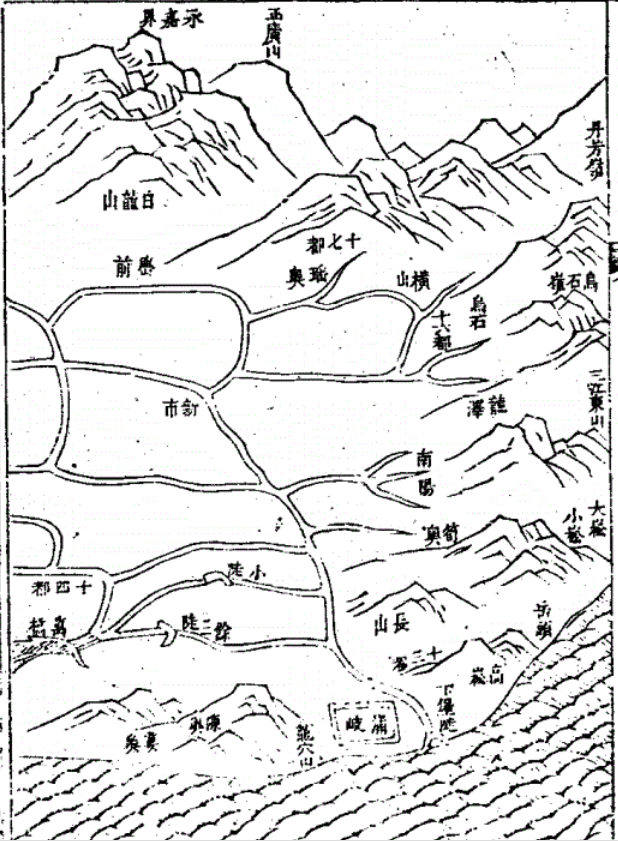

Supplement: S3 File — (ZIP) [file pone.0250622.s003.zip › S1 Scanned version of local chornicles/微信图片_20200324153605.png]

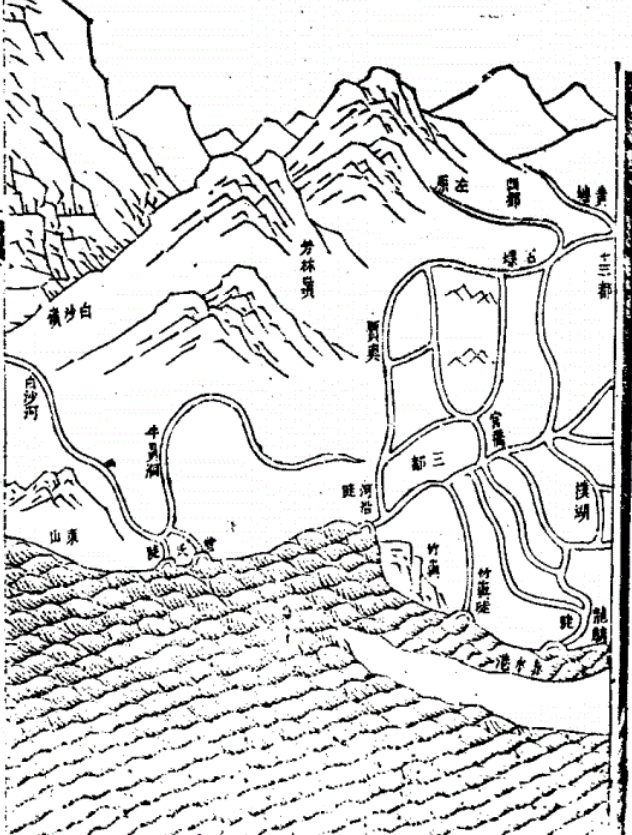

Supplement: S3 File — (ZIP) [file pone.0250622.s003.zip › S1 Scanned version of local chornicles/微信图片_20200324153609.png]

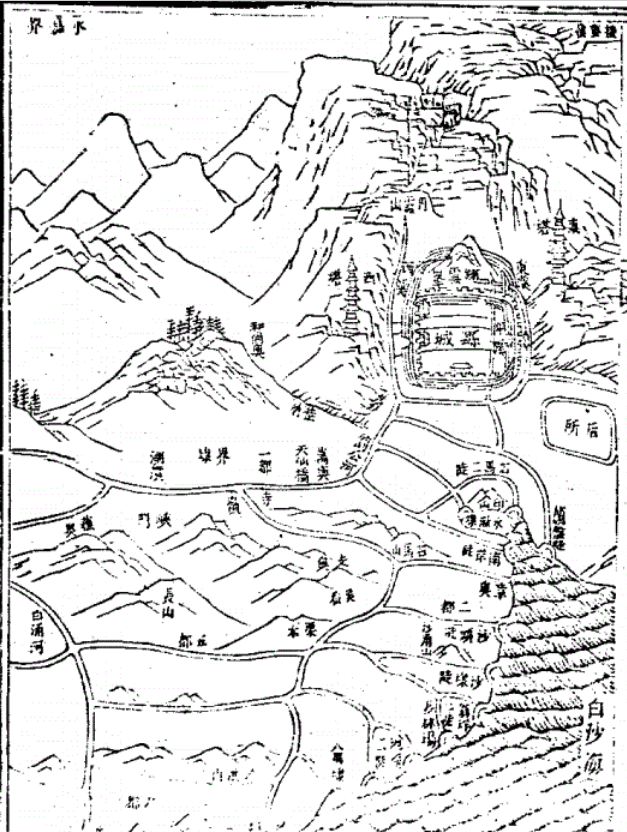

Supplement: S3 File — (ZIP) [file pone.0250622.s003.zip › S1 Scanned version of local chornicles/微信图片_20200324153612.png]

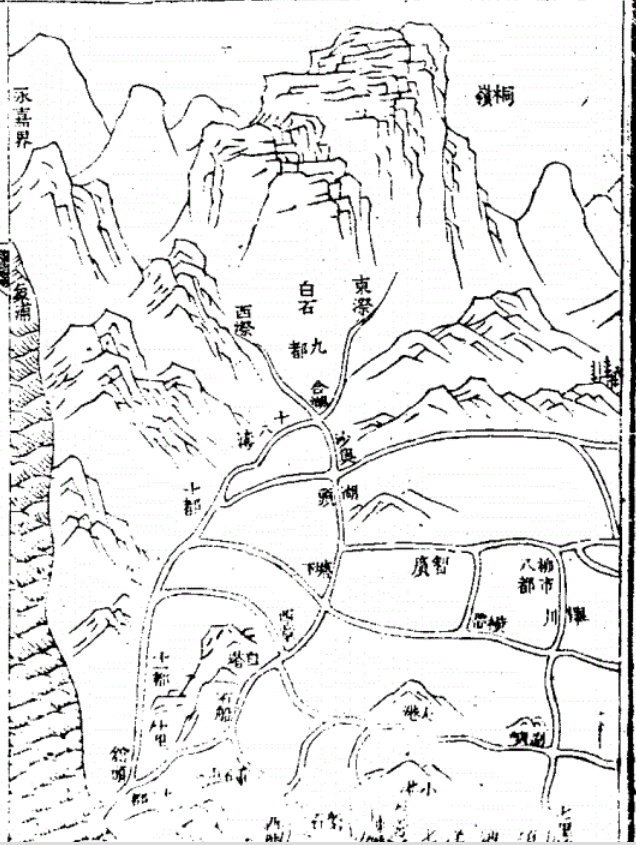

Supplement: S3 File — (ZIP) [file pone.0250622.s003.zip › S1 Scanned version of local chornicles/微信图片_20200324153615.png]

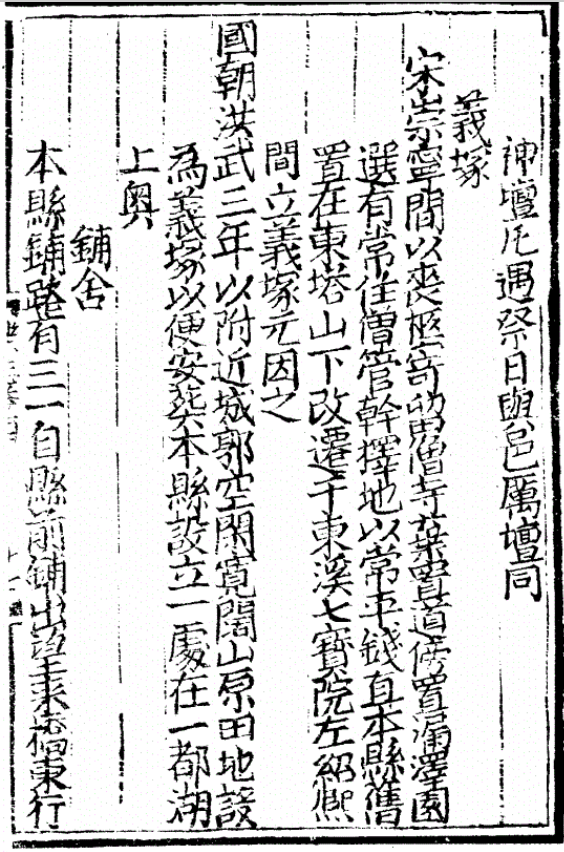

Supplement: S3 File — (ZIP) [file pone.0250622.s003.zip › S1 Scanned version of local chornicles/微信图片_20200324153619.png]

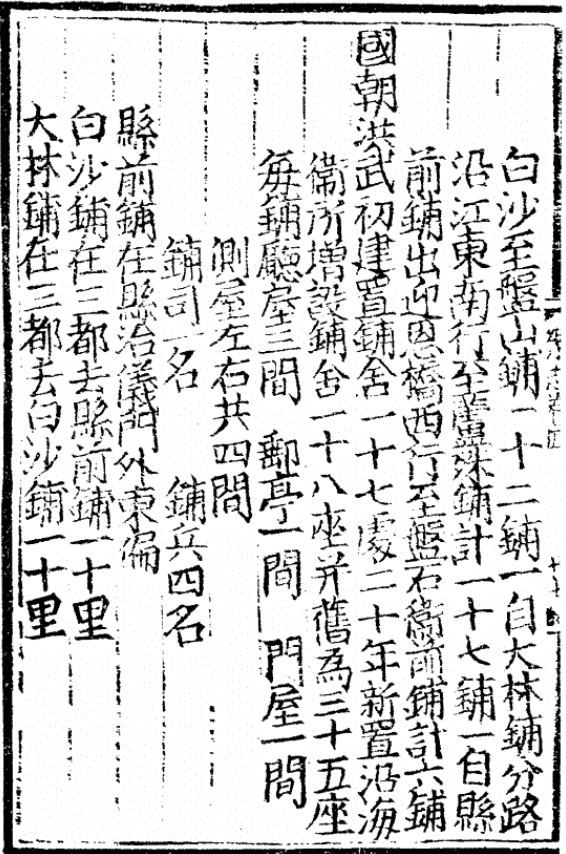

Supplement: S3 File — (ZIP) [file pone.0250622.s003.zip › S1 Scanned version of local chornicles/微信图片_20200324153623.png]

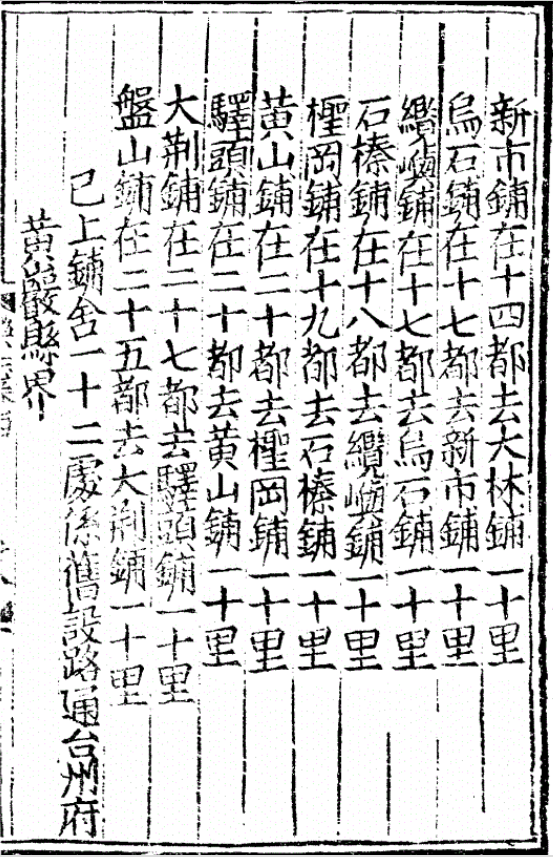

Supplement: S3 File — (ZIP) [file pone.0250622.s003.zip › S1 Scanned version of local chornicles/微信图片_20200324153627.png]

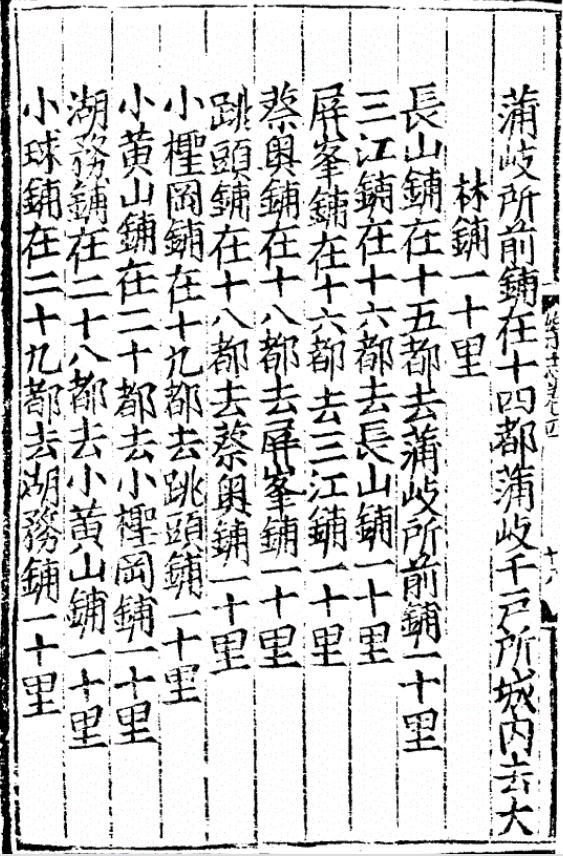

Supplement: S3 File — (ZIP) [file pone.0250622.s003.zip › S1 Scanned version of local chornicles/微信图片_20200324153639.png]

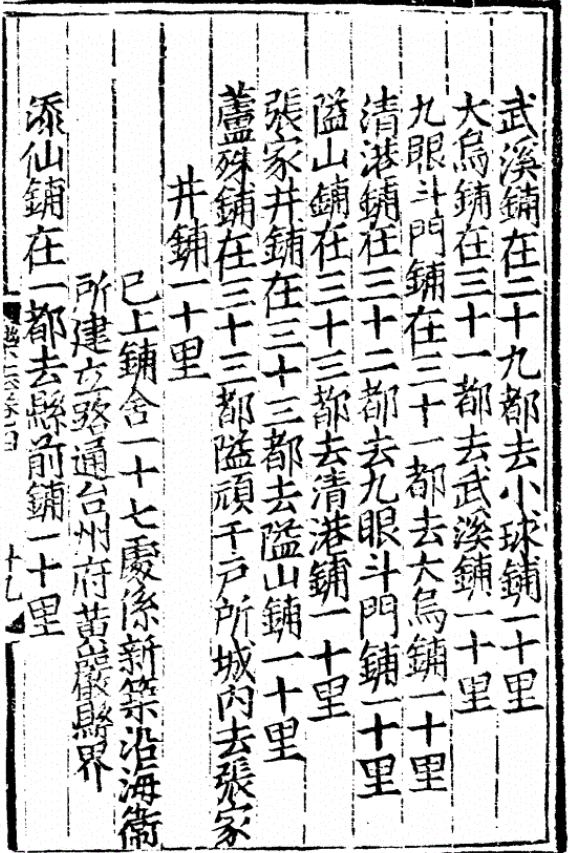

Supplement: S3 File — (ZIP) [file pone.0250622.s003.zip › S1 Scanned version of local chornicles/微信图片_20200324153642.png]

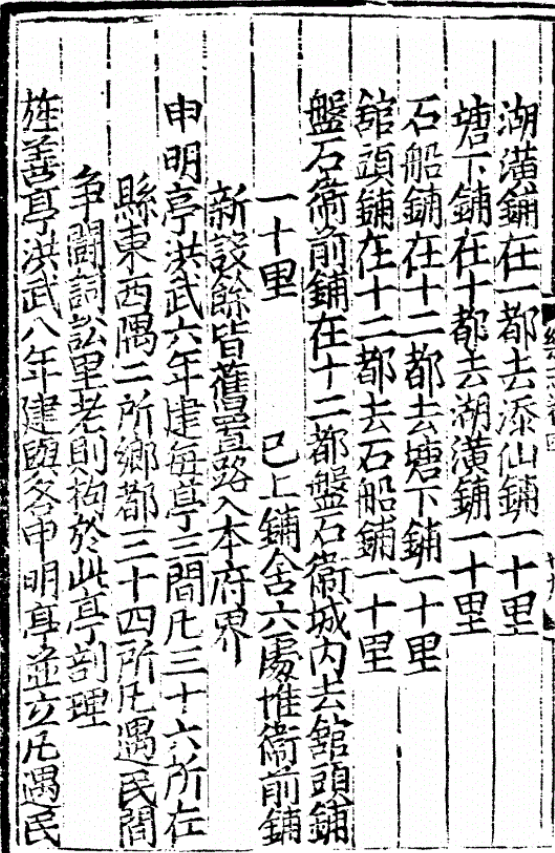

Supplement: S3 File — (ZIP) [file pone.0250622.s003.zip › S1 Scanned version of local chornicles/微信图片_20200324153646.png]

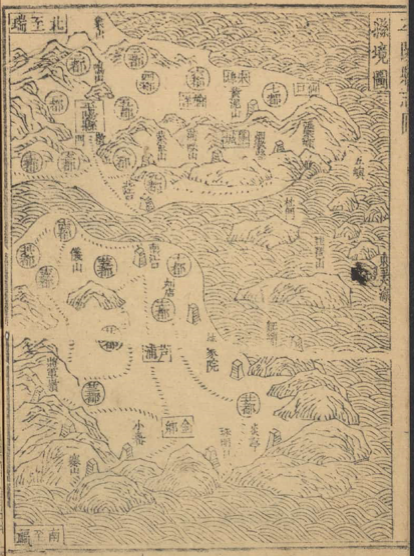

Supplement: S3 File — (ZIP) [file pone.0250622.s003.zip › S1 Scanned version of local chornicles/微信图片_20200324153654.png]

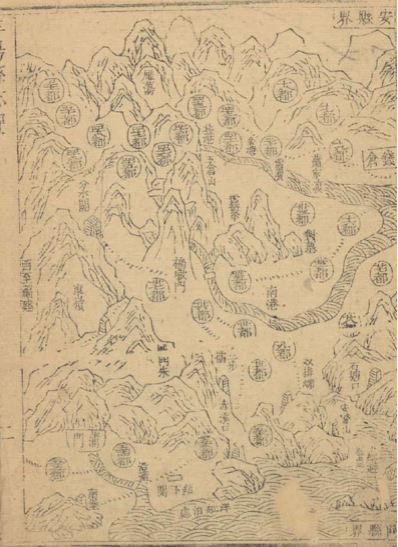

Supplement: S3 File — (ZIP) [file pone.0250622.s003.zip › S1 Scanned version of local chornicles/微信图片_20200324153657.png]

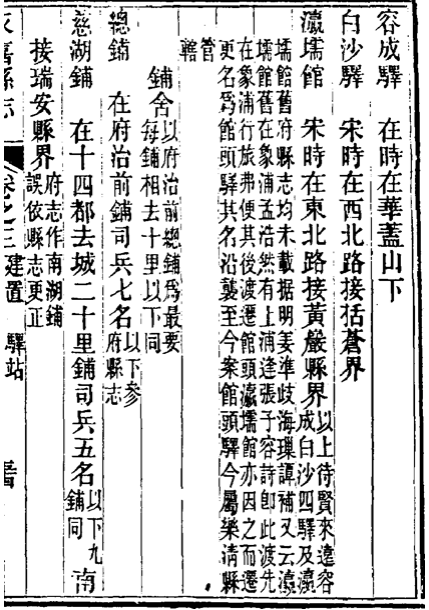

Supplement: S3 File — (ZIP) [file pone.0250622.s003.zip › S1 Scanned version of local chornicles/微信图片_20200324153701.png]

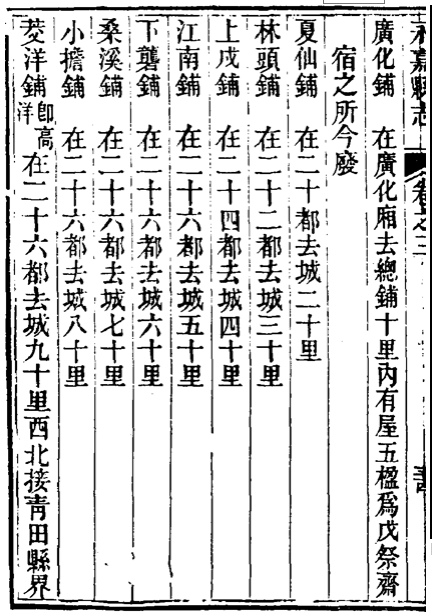

Supplement: S3 File — (ZIP) [file pone.0250622.s003.zip › S1 Scanned version of local chornicles/微信图片_20200324153705.png]

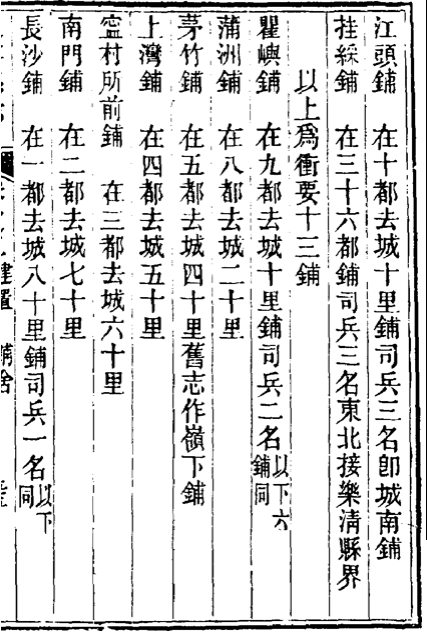

Supplement: S3 File — (ZIP) [file pone.0250622.s003.zip › S1 Scanned version of local chornicles/微信图片_20200324153709.png]

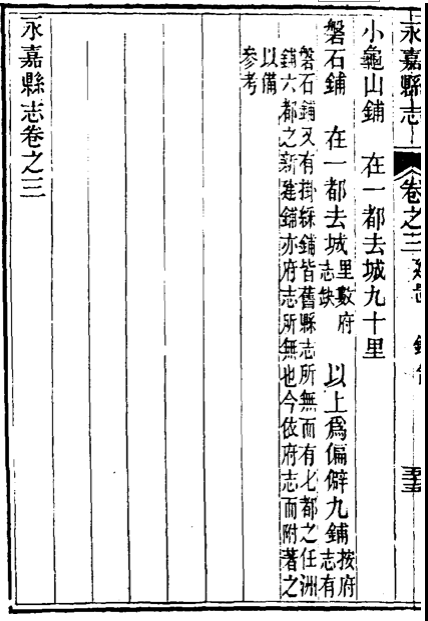

Supplement: S3 File — (ZIP) [file pone.0250622.s003.zip › S1 Scanned version of local chornicles/微信图片_20200324153713.png]

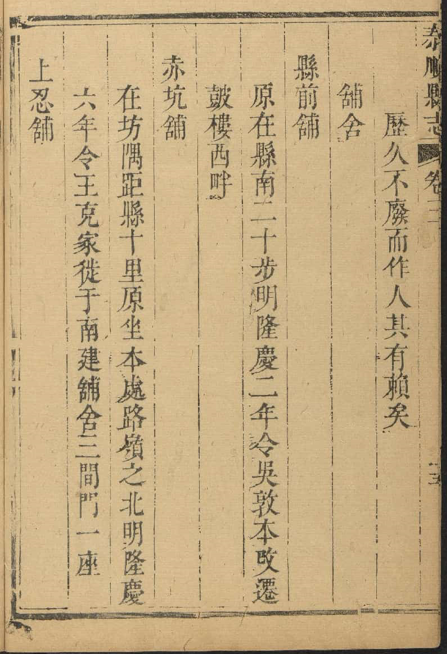

Supplement: S3 File — (ZIP) [file pone.0250622.s003.zip › S1 Scanned version of local chornicles/泰顺县志1.png]

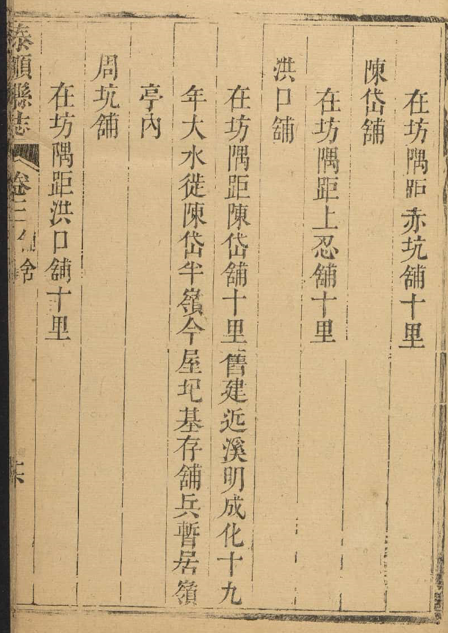

Supplement: S3 File — (ZIP) [file pone.0250622.s003.zip › S1 Scanned version of local chornicles/泰顺县志2.png]

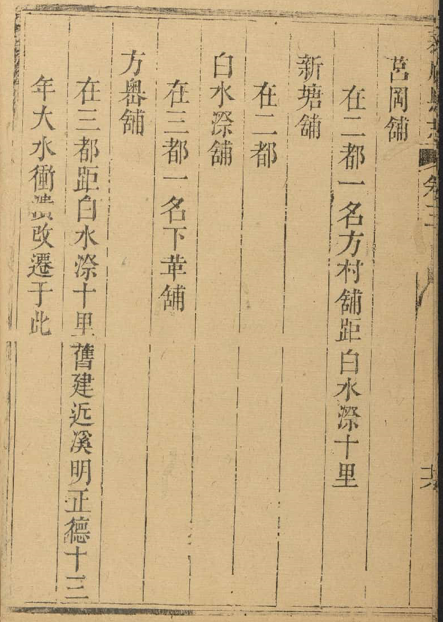

Supplement: S3 File — (ZIP) [file pone.0250622.s003.zip › S1 Scanned version of local chornicles/泰顺县志3.png]

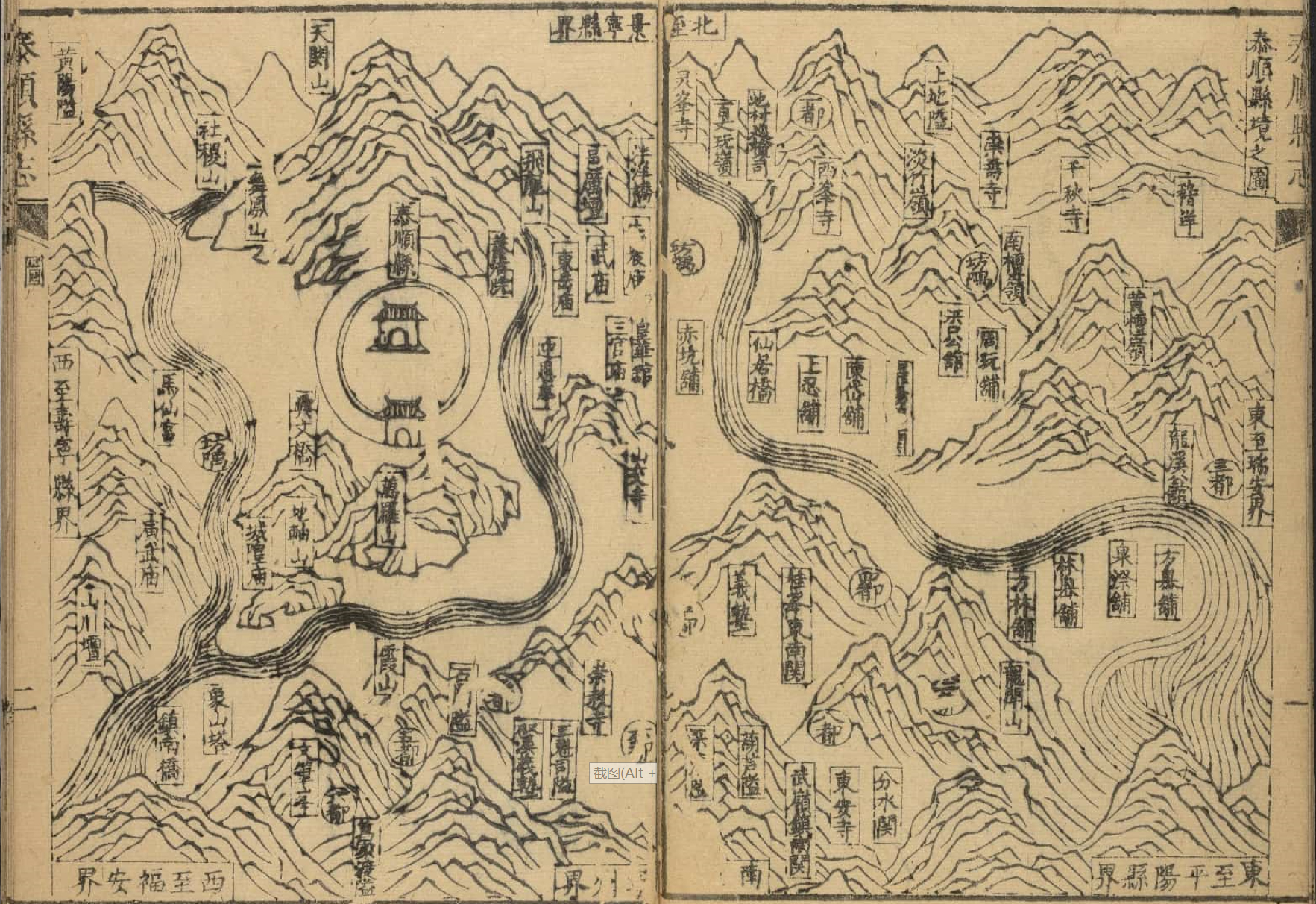

Supplement: S3 File — (ZIP) [file pone.0250622.s003.zip › S1 Scanned version of local chornicles/泰顺县舆图.jpg]
