# Supplementary material for: Quantitative research on the efficiency of ancient information transmission system: A case study of Wenzhou in the Ming Dynasty
Source: PLoS One. 2021 Apr 23;16(4):e0250622. doi: 10.1371/journal.pone.0250622 (PMC8064551; doi:10.1371/journal.pone.0250622)
Supplement: S4 File — (ZIP) [file pone.0250622.s004.zip › S4 Average Nearest Neighbor calculation results/Calculation result of Pingyang‘s Urgent Delivery Stations.html]

xml version='1.0' encoding='UTF-8'?
 平均最近邻汇总

# 平均最近邻汇总

|  |  |  |
| --- | --- | --- |
| 最邻近比率: | 1.134225 |  |
| z 得分: | 1.406453 |  |
| p 值: | 0.159590 |  |

z 得分为 1.40645254253， 该模式与随机模式之间的差异似乎并不显著。

平均最近邻汇总

| 平均观测距离: | 3850.1997 Meters |
| 预期平均距离: | 3394.5642 Meters |
| 最邻近比率: | 1.134225 |
| z 得分: | 1.406453 |
| p 值: | 0.159590 |

数据集信息

| 输入要素类: | 平阳县急递铺 |
| 距离法: | EUCLIDEAN |
| 研究区域: | 1382767915.874176 |
| 选择集: | False |
