# Supplementary material for: Quantitative research on the efficiency of ancient information transmission system: A case study of Wenzhou in the Ming Dynasty
Source: PLoS One. 2021 Apr 23;16(4):e0250622. doi: 10.1371/journal.pone.0250622 (PMC8064551; doi:10.1371/journal.pone.0250622)
Supplement: Attachment — Submitted filename: Response to Reviewers.docx [file pone.0250622.s006.docx]

Dear editor and reviewer,

On behalf of all the contributing authors, I would like to express our sincere appreciations of your letter and reviewers’ constructive comments concerning our article entitled “**Quantitative research on the efficiency of ancient information transmission system：A case study of Wenzhou in the Ming Dynasty**” (Manuscript No.:**PONE-D-20-35409**). These comments are all valuable and helpful for improving our article. According to the academic editor and reviewer’s comments, we have made extensive modifications to our manuscript and supplemented extra data to make our results convincing. In the revised version, changes to our manuscript were all highlighted within the document by using “Track Changes”. For the places that not been modified according to the comments, we also made explanations. Point-by-point responses to the academic editor and reviewer are listed below this letter.

**Response to academic editor’s comments:**

1) Please ensure that your manuscript meets PLOS ONE's style requirements, including those for file naming.

Response: We modified the manuscript according to the formatting sample of PLOS ONE to ensure that all formats and file naming have been adjusted to conform to the requirements indicated in PLOS ONE's submission guidelines.

2) We note that Figure(s) S1, 1, 3, 5, 6 and 7 in your submission contain map images which may be copyrighted....We require you to either (1) present written permission from the copyright holder to publish these figures specifically under the CC BY 4.0 license, or (2) remove the figures from your submission.

Response: We are very sorry for our negligence of the copyright issue in the process of using the pictures. Some of the pictures mentioned above are self drawn and there is no copyright licensing problem. Others are removed or replaced according to the suggestions.

Figure S1 is not directly related to the manuscript, we just take it as a reference material. In order to avoid unnecessary misunderstanding and copyright disputes, we removed Figure S1 and add the atlas with Figure S1 (Qixiang Tan. The Historical Atlas of China. Sino Maps Press; 1982.) into the reference.

Figure 1 contains two maps, one is the location of Wenzhou in the Ming Dynasty, the other is the administrative map of Wenzhou. Unfortunately, we didn't get in touch with the author of the Ming Dynasty map, so we have to replace this map. The new map of China is provided by Ministry of Natural Resources of China (http://bzdt.ch.mnr.gov.cn). According to the website, the public can browse and download the standard map for free, and the map approval number should be marked when using the standard map directly. We use this map as required. While the administrative map of Wenzhou remains, because it is self drawn with reference to historical materials.

Figure(s) 3, 5, 6 and 7 in the manuscript are self drawn by using the ArcGIS software. All the information contained in these maps are collected from the historical materials, and analyzed by the spatial tools of ArcGIS software.

**Response to reviewer’s comments:**

1) Although the authors allude to Chinese antiquity and advance the idea that, compared to previous systems, the information transmission systems under the Ming were '' very developed '' (line 45), this claim is not proven, especially compared to those of Yuan, Song and Tang Dynasties.

Response: The transmission system of Ming Dynasty inherited from that of the Yuan Dynasty. Compared with the transmission system of the Yuan, Song and Tang Dynasties, the laws on the transmission system in the Ming Dynasty were more completed, which had made specific provisions on the organization, network distribution, mileage time limit and funding quota. In addition, the post roads in the border areas were hewed out, which made the coverage of the transmission system larger. (see Guangsheng Liu, Meizhuang Zhao. The history of ancient post in China. Posts and Telecommunications Press; 1999.) Therefore, the transmission system of the Ming Dynasty was "very developed". We are very sorry that our expression is not rigorous enough, so we make a supplementary explanation about the development of the transmission system in the Introduction part.

2) We are thus surprised to read line 40 that “In ancient China, where technology was underdeveloped...”! We do not agree, on the contrary, technologies in China were very developed for those times! This is what the research and work of J. Needham and his collaborators has demonstrated.

Response: Thank the reviewer for pointing out this wrong expression. Our original intention of “underdeveloped” refers to the comparison with the convenient and diversified communication tools in modern society. In order to emphasize that the dissemination of information in ancient China could only rely on transmission system built by the government. But it does cause ambiguity, so we delete the modification of “where technology was underdeveloped”.

3) It is regrettable that, despite a rich bibliography, no mention is made of works relating to the history of the Chinese postal systems which shows a continuity of this organisation from antiquity to pre-modern times (see Gazagnadou, Didier, 2016, The diffusion of a postal relay system in Premodern Eurasia, Kimé ed., Paris, chap. I and II and the bibliography).

Response: We sincerely appreciate the valuable comments. As suggested by the reviewer, we have read more documents on the history of Chinese postal system and made a supplement of that in the Introduction part to support this study.

4) As for the efficiency of the system in the transmission of information, the authors should have mentioned the speed of circulation of postal mails to link the towns together (Gazagnadou, chap. I) and that, for the land post, the efficiency of the transmission depends on the type of horses and the number of kilometres separating the different relays (Minetti, Alberto, 2003, Efficiency of the Equine of the postal system, Nature, n ° 246, December) and of course on the regional topography. The authors could have provided a map and idea of the area covered by postal routes equipped with the different types of relay and post stations.

Response: The land information transmission in Ming Dynasty could be divided into two ways, one was the urgent delivery stations relying on couriers to deliver common documents by walking, and the other was the post stations equipped with horses, boats or sedan chairs. In Wenzhou, where the waterway was deveoped, post route was connected by land and water. Post stations were equipped with boats to deliver, or sedan chairs for escorting ambassadors. (see the local chronicles of Wenzhou) We appreciate Minetti and Alberto's research on post horses. They contributed valuable information to the research of postal system. But their findings do not suit for this study, because there was no post horse in Wenzhou. As can be seen from the post equipment, Wenzhou’s land transmission was mainly relied on the urgent delivery stations. If we understand correctly, the reviewer's suggestion is to take into account the impact of post equipment on the study of transmission efficiency. We totally agree with that. Post equipment is indeed a factor which we ignored. Therefore, we have supplemented the data on the equipment of post stations and urgent delivery stations, that is, the number of couriers and boats, and rebuilt the evaluation model, to make the results more rigorous and convincing. In addition, according to the reviewer's suggestion, we also reconstructed the historical map of Wenzhou’s transmission system by adding the postal routes.

5) This article with very interesting materials and perspectives should be enriched by more technical information, in particular on the '' beacon tower (烽燧, Fēng suì) '', the '' post station (驿, Yì) '' and the '' urgent delivery station (急递铺, Jí dì pù) '' and this should be possible in particular by making full use of all the information contained in the local chronicles of Wenzhou from the Ming period that the authors quote in their tables.

Response: Thank the reviewer for the affirmation of this study. In the part of Study object, we supplement the technical information about beacon towers, post stations and urgent delivery stations, especially their transmission mode and organization mode, to support the following discussion.

6) Finally, the authors do not insist enough the links between the transmission of the military information and the politics and the pivotal role of this system of transmission of information and the crucial role of horse in this organisation for the efficiency of the system.

Response: In the Discussion part, we reanalyzed Wenzhou’s information transmission system and discussed the relationship between the information transmission and military and politics, as well as the pivotal role of the transmission of information system. As previously stated, Wenzhou’s post station did not use horse as transmission tool, therefore, we do not insist the role of horse.

I hope that the changes and explanations we’ve made resolve all your concerns about the article. I’m more than happy to make any further changes that will improve the paper and facilitate successful publication. Thank you very much for your help.

Sincerely,

Bei Wu
